# Structural basis of Zn(II) induced metal detoxification and antibiotic resistance by histidine kinase CzcS in *Pseudomonas aeruginosa*

**DOI:** 10.1371/journal.ppat.1006533

**Published:** 2017-07-21

**Authors:** Dan Wang, Weizhong Chen, Shanqing Huang, Yafeng He, Xichun Liu, Qingyuan Hu, Tianbiao Wei, Hong Sang, Jianhua Gan, Hao Chen

**Affiliations:** 1 Coordination Chemistry Institute and the State Key Laboratory of Coordination Chemistry, School of Chemistry and Chemical Engineering, Collaborative Innovation Center of Chemistry for Life Sciences, Nanjing University, Nanjing, P.R. China; 2 Jinling Hospital, Department of Dermatology, Medical School of Nanjing University, Nanjing University, Nanjing, P. R. China; 3 State Key Laboratory of Genetic Engineering, Collaborative Innovation Center of Genetics and Development, Department of Physiology and Biophysics, School of Life Sciences, Fudan University, Shanghai, China; Children's Hospital Boston, UNITED STATES

## Abstract

*Pseudomonas aeruginosa* (*P*. *aeruginosa*) is a major opportunistic human pathogen, causing serious nosocomial infections among immunocompromised patients by multi-determinant virulence and high antibiotic resistance. The CzcR-CzcS signal transduction system in *P*. *aeruginosa* is primarily involved in metal detoxification and antibiotic resistance through co-regulating cross-resistance between Zn(II) and carbapenem antibiotics. Although the intracellular regulatory pathway is well-established, the mechanism by which extracellular sensor domain of histidine kinase (HK) CzcS responds to Zn(II) stimulus to trigger downstream signal transduction remains unclear. Here we determined the crystal structure of the CzcS sensor domain (CzcS SD) in complex with Zn(II) at 1.7 Å resolution. This is the first three-dimensional structural view of Zn(II)-sensor domain of the two-component system (TCS). The CzcS SD is of α/β-fold in nature, and it senses the Zn(II) stimulus at micromole level in a tetrahedral geometry through its symmetry-related residues (His55 and Asp60) on the dimer interface. Though the CzcS SD resembles the PhoQ-DcuS-CitA (PDC) superfamily member, it interacts with the effector in a novel domain with the N-terminal α-helices rather than the conserved β-sheets pocket. The dimerization of the N-terminal H1 and H1’ α-helices is of primary importance for the activity of HK CzcS. This study provides preliminary insight into the molecular mechanism of Zn(II) sensing and signaling transduction by the HK CzcS, which will be beneficial to understand how the pathogen *P*. *aeruginosa* resists to high levels of heavy metals and antimicrobial agents.

## Author summary

*P*. *aeruginosa* inhabits diverse environments and is one of the most prevalent opportunistic human pathogens of immunocompromised patients. The high antibiotic resistance is a major cause of therapeutic failure in the treatment of *P*. *aeruginosa* infections. The opportunistic pathogen *P*. *aeruginosa* co-regulates cross-resistance between Zn(II) and carbapenem antibiotics by the CzcR-CzcS signal transduction system. The extracellular Zn(II) stimulus is sensed by the HK CzcS and further triggers metal detoxification and antibiotic resistance through intracellular regulatory pathway. Here, we provide the three-dimensional structure of CzcS SD in complex with the Zn(II). Based on the structure, several key residues for Zn(II) sensing and regulation are identified, and the signal transduction is disclosed to be modulated by the dimerization of N-terminal α-helices in the sensor domain. Our research will provide potential guidance for the treatment of clinical issues caused by co-regulation between heavy metals and antibiotics in *P*. *aeruginosa*.

## Introduction

Bacteria are extremely versatile that can regulate cellular processes in a sophisticated manner and thereby survive in changing environments. The two-component system (TCS) is the predominant strategy for coupling various extracellular stimuli to appropriate cellular responses in microorganisms [1–5]. In *Pseudomonas aeruginosa (P*. *aeruginosa)*, approximately 130 genes have been identified that encode various types of TCSs [5, 6]. These regulatory systems enable this organism to ubiquitously exist in diverse environments and to express various virulence factors [7, 8]. Thus, *P*. *aeruginosa* is one of the most prevalent opportunistic pathogens and causes severe hospital-acquired infections among immunocompromised patients [7, 9, 10]. It is capable of causing both chronic and acute pulmonary infections in cystic fibrosis (CF) patients, ventilator-associated pneumonia, and sepsis in burn patients [8]. Moreover, this pathogenic bacterium possesses intrinsically high levels of resistance to multiple classes of antimicrobial agents, presenting tremendous obstacles for anti-infective therapies [7, 11].

**Fig 1:**
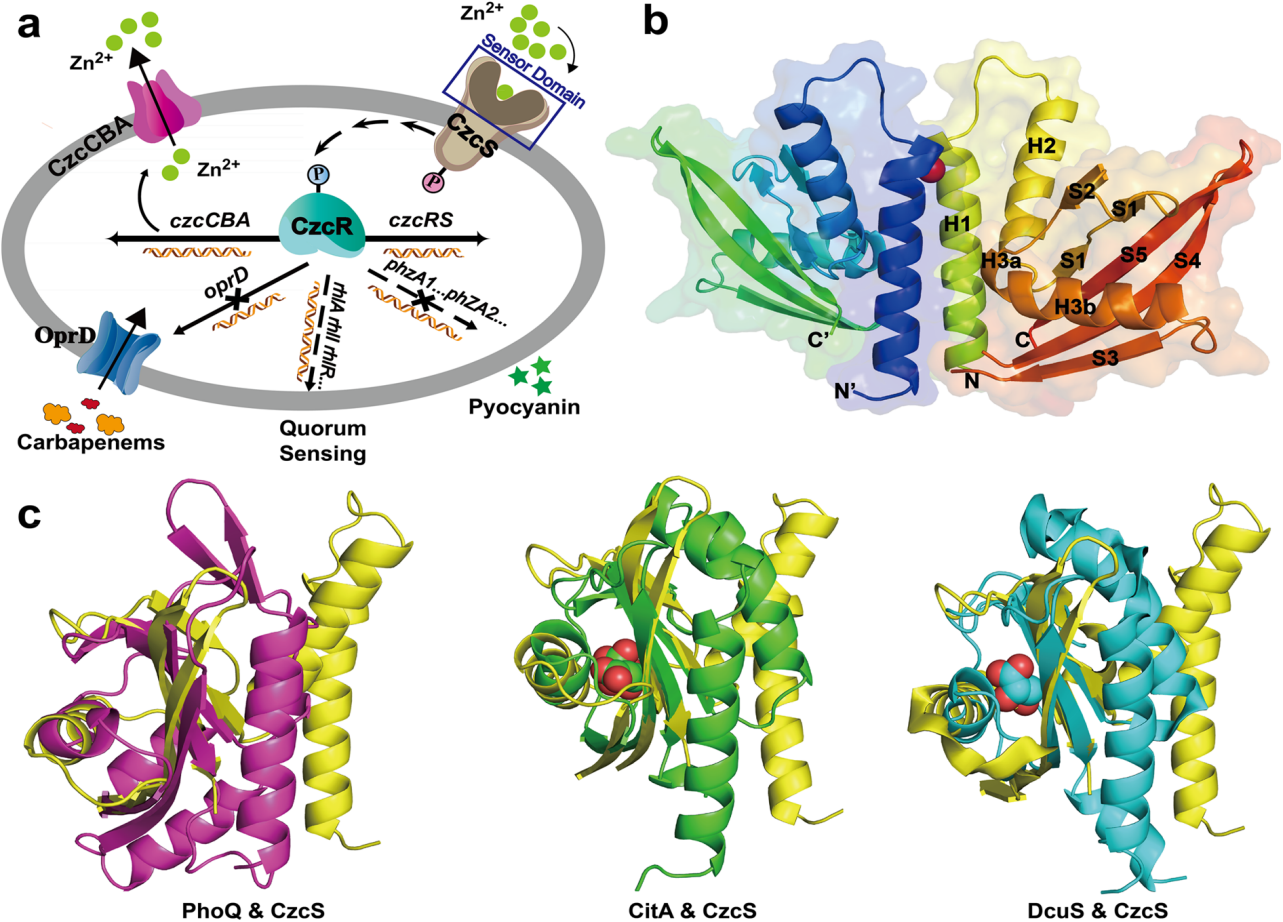
Schematic diagram of the CzcR-CzcS TCS in *P*. *aeruginosa*, overall structure of CzcS-Zn, and superimpositions of CzcS SD with other PDC members. (a) A schematic diagram of the CzcR-CzcS TCS in *P*. *aeruginosa*. The CzcR-CzcS TCS is involved in regulating Zn(II) resistance, antibiotic resistance, quorum sensing, and virulence factors. The periplasmic sensor domain of the HK CzcS is indicated with the blue box. (b) Overall structure of CzcS-Zn. The CzcS SD are shown as cartoon diagrams with labelled secondary structures. The coordinated Zn(II) is depicted as sphere in red. (c) Superimpositions of the CzcS SD with other PDC members in tertiary structure. All structures are shown as cartoon representations with the CzcS SD colored in yellow in all panels. The PhoQ SD (PDB code: 3BQ8), CitA SD (PDB code: 2J80), and DcuS SD (PDB code: 3BY8) are colored in magenta, green, and cyan, respectively. The citrate and malate ligands, which are bound to the central β-sheets, are shown as spherical representations in CitA and DcuS, respectively. The pairwise structure comparisons are performed by the PDBefold [17].

The CzcR-CzcS TCS in *P*. *aeruginosa* is responsible for numerous cellular processes, including Zn(II) resistance, carbapenem antibiotic resistance, quorum sensing, and virulence regulation **(Fig 1A)** [12–14]. Under direct stimulation by Zn(II), the histidine kinase (HK) CzcS auto-phosphorylates on its conserved histidine residue. It subsequently transmits the phosphoryl group to the conserved aspartate residue in the receiver domain of the response regulator (RR) CzcR. The phosphorylated CzcR up-regulates the expression of a metal efflux pump, CzcCBA. It also represses the expression of OprD, a porin that regulates the entry of basic amino acids and carbapenem antibiotics [13, 14]. This co-regulation between metal detoxification and antibiotic resistance is unusual, and its mechanism will provide significant guidance for the treatments of environmental and clinical issues.

In the CzcR-CzcS TCS, the HK CzcS is predicted to be the transmembrane sensor-transmitter, and it contains three functional domains [15]. The highly diverse N-terminal periplasmic sensor domain is arranged between two membrane-spanning segments and is followed by a conserved C-terminal cytoplasmic kinase domain. The stimulus is detected by the periplasmic sensor domain and transmitted across the membrane to the cytoplasmic kinase domain [3, 16]. Given the pivotal role of the extracellular sensor domain in signal recognition and transduction, we determined the crystal structure of the CzcS sensor domain (CzcS SD) in the presence of Zn(II) (referred as CzcS-Zn hereafter). Together with the biochemical and *in vivo* studies, the CzcS SD is identified to bind Zn(II) between N-terminal H1 and H1’ α-helices, which is the key first step in Zn(II) detoxification and meropenem resistance by HK CzcS. The N-terminal H1 and H1’ α-helices are also shown to play important roles in signal transduction via a series of structure-guided mutagenesis studies. The study reveals the CzcS SD appears to utilize a new mode which is not previously observed for sensor HKs to protect *P*. *aeruginosa* from high levels of Zn(II) and in parallel meropenem.

## Results

### Folding of the CzcS SD

The structure of CzcS-Zn complex was solved by single-wavelength anomalous diffraction (SAD) using the data collected at the zinc peak wavelength (1.2823 Å) and was refined using data collected at a wavelength of 1.0000 Å. The *R*_*work*_ and *R*_*free*_ are 0.210 and 0.253, respectively. The data collection and other refinement statistics are summarized in **Table 1**. The structure belongs to the C2 space group and it contains two molecules (CzcS SD, amino acids 40–166 in the CzcS protein) per asymmetric unit **(S1A Fig)**. As supported by the clear electron density, residues 40–132 and 135–161 in both molecules are well defined, whereas the C-terminal segment (residues 162–166) and the loop (residues 133–135) that connects the S4 and S5 β-strands are disordered. The tertiary structures of the two molecules are similar **(S1B Fig)**. The root-mean-square deviation (r.m.s.d.) between them is 0.8Å with 104 pairs of corresponding Cα atoms superimposed. The structural deviations are mainly caused by the tilting of the N-terminal domain (residues 40–81) with respect to the C-terminal domain (residues 83–161).

**Table 1:** Data collection and refinement statistics for the structure of CzcS-Zn.

	CzcS-Zn	
**Diffraction data**		
X-Ray source	Synchrotron	Synchrotron
Wavelength (Å)	1.2823	1.0000
Space group	C 2	C 2
Cell parameter		
a, b, c(Å)	93.2, 47.8, 57.8	93.3, 47.7, 57.5
α, β, γ(°)	90.0, 91.6, 90.0	90.0, 91.6, 90.0
Resolution (Å)*	30–2.33 (2.33–2.42)	30–1.70 (1.76–1.70)
R_sym_ (%) *	8.2 (13.5)	6.7 (30.0)
Completeness (%)*	95.0 (98.6)	94.5 (92.6)
I/σ(I) *	49.5 (35.0)	21.8 (2.2)
Redundancy*	6.7 (7.3)	5.2 (3.9)
**Refinement**		
Refinement program		PHENIX
Resolution (Å)		28.8–1.70
No. unique reflections		26314
R _work_ / R _free_ (%)		21.0 /25.4
No. atoms		
Protein		1859
Zn^2+^		2
Water		233
B-factors		
Protein		27.6
Ligand		24.5
Water		34.5
Ramachandran plot**		
Favored (%)		98.8
Allowed (%)		1.2
Disallowed (%)		0
**Protein Data Bank code**		5GPO

*Highest-resolution shell is shown in parentheses.

**Ramachandran plot was calculated using RAMPAGE in the CCP4 suite.

The structure of CzcS SD is a mixed α/β-fold in nature, which can be divided into two domains **(Fig 1B)**. The N-terminal helix-loop-helix domain is composed of the H1 α-helix (residues 40–60), the connecting loop (residues 61–66), and the H2 α-helix (residues 67–81). It is connected to the C-terminal domain by one residue, Thr82. The C-terminal domain contains five β-strands (S1-S5) and one 03B1-helix (H3). The five β-strands form one anti-parallel β-sheet. S5 (residues 150–161) is located in the middle and is flanked by S1 (residues 83–90) and S2 (residues 97–102) on one side and by S4 (residues 136–147) and S3 (residues 126–132) on the other side. The H3 α-helix packs against the anti-parallel β-sheet and is nearly perpendicular to the first two α-helices. A short kink at residues 106 and 107 divides the H3 α-helix into H3a (residues 103–105) and H3b (residues 108–120). The N-terminal H1 α-helix and the C-terminal S5 β-strand are oriented in the same direction, which connect to the transmembrane helix (TM1 and TM2 helices) in the transmembrane domain of HK CzcS.

### Structural similarities of CzcS SD with other PDC superfamily members

The typical PDC members include the divalent cation sensor PhoQ [18, 19], the citrate sensor CitA [20, 21], and the C4-dicarboxylate sensor DcuS [22]. Despite of the negligible sequences identities, it reveals that the CzcS SD possesses a similar structural arrangement to those of other members of the PDC superfamily by the structural similarity searches performed with the Dali server program [23] **(S2 Fig)**. The relatively high Z-scores of 5.4, 4.0, and 7.0 are yielded in the structure-based alignments of the CzcS SD with PhoQ [18], CitA [20], and DcuS [22], respectively. The sensor domain of CzcS and PhoQ can be largely superimposed in the C-terminal domain with a r.m.s.d. of 2.6 Å over 66 corresponding Cα atoms. Similarly, the CitA superimposes onto CzcS over 58 corresponding Cα positions with a r.m.s.d. of 2.9 Å, and DcuS superimposes onto CzcS over 54 corresponding Cα positions with a r.m.s.d. of 2.6 Å. The distinct difference between the structure of CzcS SD and those of the other PDC superfamily members is the orientation of the N-terminal helix-loop-helix domain **(Fig 1C)**. In the structure of CzcS-Zn, the N-terminal helix-loop-helix domain is tilted away from the central five anti-parallel β-sheet, which may be caused by the Zn(II) binding at the H1 and H1’ α-helices.

### Zn(II) binding site in CzcS SD

Two Zn(II) ions are captured in the structure of CzcS-Zn. One of the Zn(II) ions is coordinated with His72 and Asp76 of CzcS molecule A **(S1A Fig, gray)**. It is also coordinated with Asp62 of symmetry-related molecule B and His72 of symmetry-related molecule C **(S3 Fig)**. The *P*. *aeruginosa* functions normally in response to extracellular Zn(II) with the double mutation of His72 and Asp76 on HK CzcS **(S4 Fig)**. The result shows that this Zn(II) binding pattern is physiologically irrelevant and may be caused by crystallographic packing.

**Fig 2:**
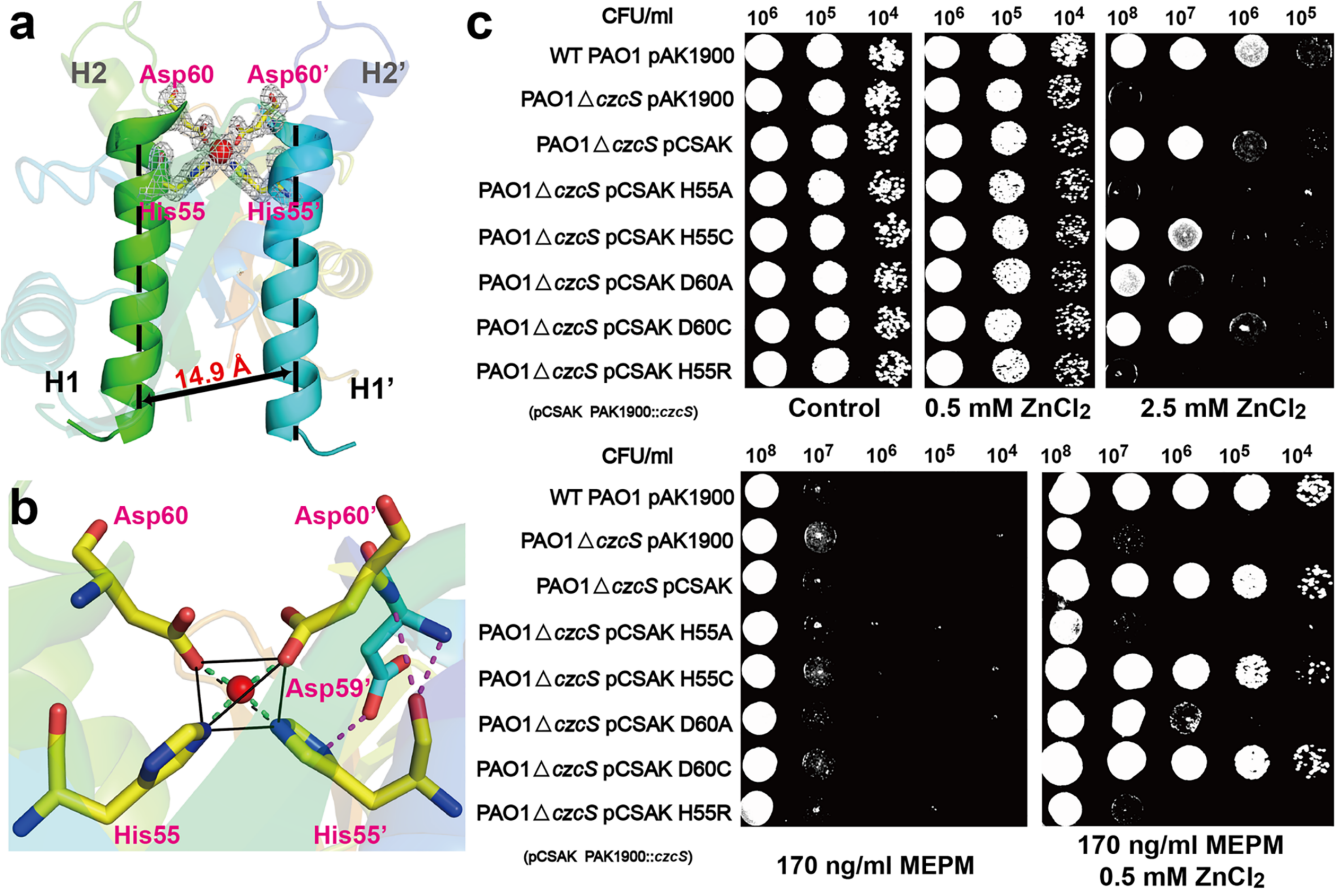
The Zn(II) binding site of CzcS SD. (a) The coordination environment of the functionally relevant Zn(II) ion. The Zn(II) ion is shown as sphere in red, and the coordinated residues are shown as sticks in atomic color (C, yellow; N, blue; O, red). The 2F_o_-F_c_ omit map is contoured at the 1σ level and is colored in gray. (b) The detailed interactions between the Zn(II) and the coordinated residues. The Zn(II) binds to CzcS in the tetrahedral coordination geometry. The Zn(II) ion is shown as red sphere. The residues in the first coordination sphere are depicted as stick representations with Cα atoms in yellow. The second shell hydrogen-bond interactions are indicated by the magenta-dotted line. (c) Metal and antibiotic tolerance plate assay. Wild type *P*. *aeruginosa* and its derivative strains are examined on LB plates that contain Zn(II) and MEPM antibiotic as follows: wild type *P*. *aeruginosa* with empty pAK1900 plasmid as the control (WT PAO1 pAK1900), *czcS*-deficient *P*. *aeruginosa* with the empty pAK1900 (PAO1△*czcS* pAK1900), *czcS*-deficient *P*. *aeruginosa* with wild type *czcS* encoded on pAK1900 (PAO1△*czcS* pCSAK), *czcS*-deficient *P*. *aeruginosa* complemented with the *czcS* mutants in pAK1900 (PAO1△*czcS* pCSAK H55A, PAO1△*czcS* pCSAK H55C, PAO1△*czcS* pCSAK D60A, PAO1△*czcS* pCSAK D60C, and PAO1△*czcS* pCSAK H55R).

The other Zn(II) is identified to be functional relevant and is associated with the second CzcS molecule **(S1A Fig, green)** shown in the asymmetric unit. Double mutation of coordinated residues (His55 and Asp60) of second Zn(II) on HK CzcS causes severely defects on the regulation of Zn(II) resistance in *P*. *aeruginosa* **(S4 Fig)**. In the symmetry operation, this CzcS molecule can form a homodimer **(Fig 1B)**, and the Zn(II) is exclusively buried between the central parallel H1 and H1’ α-helices which constitute the dimer interface **(Fig 2A)**. The H1 and H1’ α-helices are surrounded by multiple solvent water molecules, which facilitate the Zn(II) access to the active site. A distorted tetrahedral geometry is adopted by Zn(II) to coordinate with the symmetric ligands (His55/Asp60 and His55’/Asp60) from the H1 and H1’ α-helices, respectively **(Fig 2B)**. The His55 and His55’ residues interact with the Zn(II) through their Nε2 nitrogen atoms, and Asp60 and Asp60’ residues contact Zn(II) through the Oδ2 atoms of their carboxylate side-chain **(Fig 2B)**. The bond distances of the coordination center are 1.92 Å-2.17 Å with the bond angles ranging from 102.0° to 127.6°. In each monomer, the Oδ2 atom of Asp59 residue makes a hydrogen-bond interaction with the Nδ1 nitrogen atom of the His55 residue. Additionally, the O atom of His55 forms hydrogen-bond with the N atoms of Asp59 and Asp60, respectively **(Fig 2B)**. These second shell interactions, particularly the carboxylate side-chain with the histidine ligand, are thought to play an important role in the stability of the coordination structure [24].

### *In vivo* identification of Zn(II) binding site in HK CzcS

In view of the co-regulation of cross-resistance between metal ions and carbapenem antibiotics by CzcR-CzcS TCS, the physiological importance of the His55 and Asp60 residues in *P*. *aeruginos*a is investigated by using the Zn(II) and meropenem (MEPM) antibiotic tolerance assay **(Fig 2C)**. All the strains keep consistent growth state on the LB solid medium in the absence of Zn(II), and their growing status are not influenced until the concentration of Zn(II) reaches 0.5 mM. However, when a higher concentration of Zn(II) (2.5 mM) are supplied, the *P*. *aeruginosa* PAO1 strain demonstrates an obvious growth advantage over the *czcS-*deficient strain. The abolished metal resistance of the *czcS*-deficient strain is restored to wild type levels by complementation with a plasmid (pAK1900) carrying the *czcS* gene. When His55 and Asp60 are substituted by the amino acids which can’t coordinate with Zn(II), the mutant strains (H55A, D60A, and H55R) dramatically attenuate their abilities in the Zn(II) detoxification for the destruction of Zn(II) binding site. Remarkably, the losses in responsiveness to Zn(II) of H55A and H55R mutant strains are similar in degree to the non-responsiveness of the *czcS*-deficient strain. Additionally, the aforementioned mutant strains and the *czcS-*deficient strain lose their Zn(II)-inducible resistance to the MEPM antibiotic. The mutagenesis analyses corroborate the crucial role of the His55 and Asp60 in Zn(II) sensing. Intriguingly, markedly different phenotypes are observed when the His55 and Asp60 are replaced with the coordinated cysteine residues **(Fig 2C)**. The H55C mutant partially preserves the ability in Zn(II) detoxification and in parallel meropenem resistance. By contrast, the D60C mutant shows equivalent responsiveness to Zn(II) and MEPM to that of the wild type PAO1.

### The Co(II)-responsive mutant strain of *P*. *aeruginosa*

**Fig 3:**
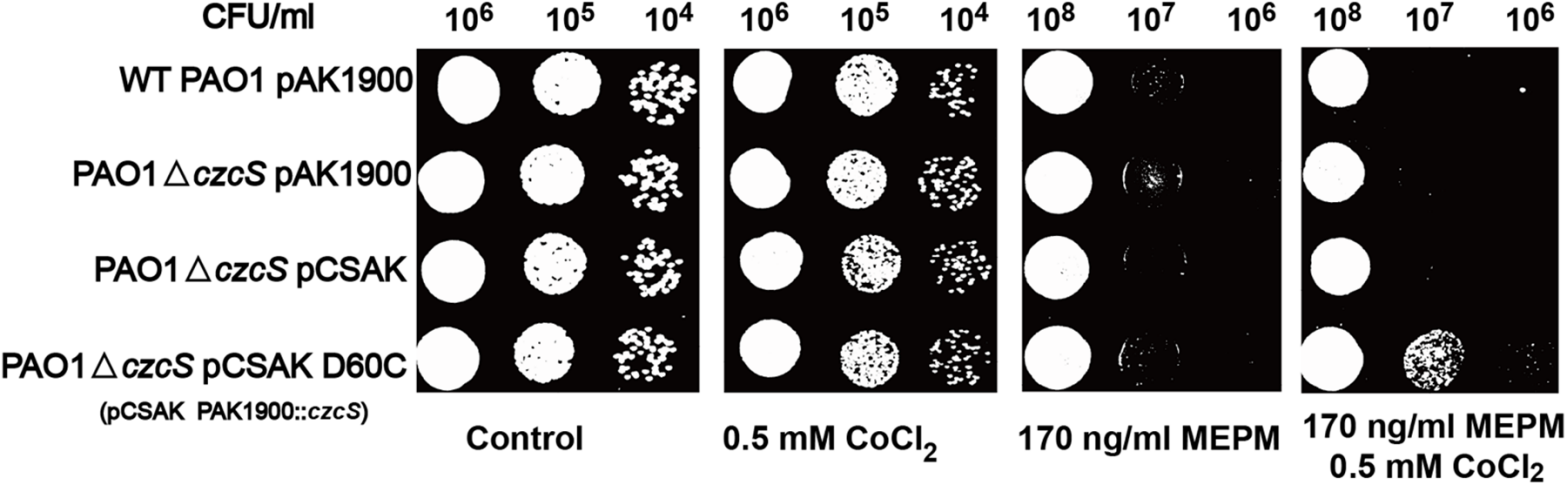
The identification of Co(II)-responsive mutant. Wild type *P*. *aeruginosa* and its derivative strains are examined on LB plates that contain Co(II) and MEPM antibiotic as follows: wild type *P*. *aeruginosa* with empty pAK1900 plasmid as the control (WT PAO1 pAK1900), *czcS*-deficient *P*. *aeruginosa* with the empty pAK1900 (PAO1△*czcS* pAK1900), *czcS*-deficient *P*. *aeruginosa* with wild type *czcS* encoded on pAK1900 (PAO1△*czcS* pCSAK), *czcS*-deficient *P*. *aeruginosa* complemented with the *czcS* mutants in pAK1900 (PAO1△*czcS* pCSAK D60C).

The Co(II) has similar radius to Zn(II), and it can bind to Cys_2_His_2_ coordination site in a tetrahedral geometry as well [25]. The wild type *P*. *aeruginosa* is blind to Co(II) that none Co(II)-inducible resistance to the MEPM antibiotic can be observed (**Fig 3**). With the mutation of residues (Asp60 and Asp60’) on the N-terminal H1 and H1’ α-helices, the D60C mutant strain displays Co(II)-inducible resistance to the MEPM antibiotic (**Fig 3**). Although the increased antibiotic resistance induced by Co(II) is not as strong as that of Zn(II), this experiment indicates that the binding of Co(II) between the N-terminal H1 and H1’ α-helices can also regulate the downstream signaling transduction in CzcR-CzcS TCS.

### Cysteine substitution of residues in the linker region of HK CzcS

**Fig 4:**
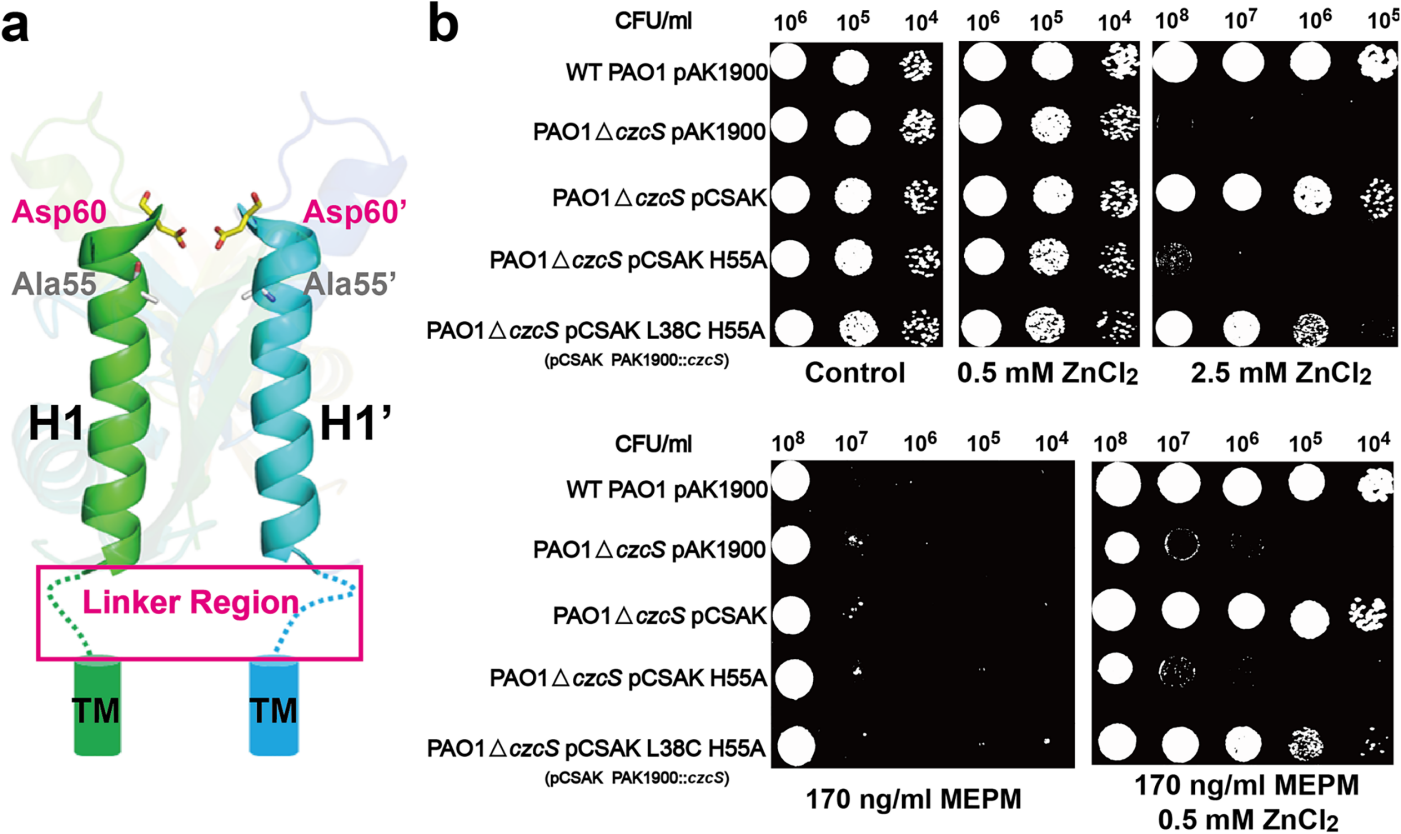
Cysteine substitution of residues in the linker region. (a) The schematic diagram of the linker region in the HK CzcS. The linker region, which connects the H1 and H1’ α-helices of the sensor domain to the transmembrane helices, is indicated by dashed lines. The amino acid sequence in the linker region is “Arg-Glu-Leu-Glu”. (b) Metal and antibiotic tolerance plate assay. Wild type *P*. *aeruginosa* and its derivative strains are examined on LB plates that contain Zn(II) and MEPM antibiotic as follows: wild type *P*. *aeruginosa* with the empty pAK1900 plasmid as the control (WT PAO1 PAK1900), *czcS*-deficient *P*. *aeruginosa* with empty pAK1900 (PAO1△*czcS* pAK1900), *czcS*-deficient *P*. *aeruginosa* with wild type *czcS* encoded on pAK1900 (PAO1△*czcS* pCSAK), *czcS* deficient *P*. *aeruginosa* complemented with *czcS* mutants in pAK1900 (PAO1△*czcS* pCSAK H55A, and PAO1△*czcS* pCSAK L38C H55A).

The linker region connects the H1 and H1’ α-helices to the transmembrane helices. It plays an important role in the process of signaling transduction from extracellular sensor domain to the transmembrane region. The cysteine substitution scanning is performed in the linker region on the basis of the H55A mutant **(Fig 4A)**. As demonstrated above, the H55A mutant loses its intrinsic resistance to Zn(II) and MEPM as the *czcS*-deficient strain due to the destruction of Zn(II) binding site. In conjunction with the mutation L38C, the L38C H55A mutant restores the responsiveness to Zn(II) stimulus. This mutant strain can survive on the LB medium with high concentrations of Zn(II) and shows Zn(II)-inducible resistance to MEPM **(Fig 4B)**. The experiment indicates that the strain with cysteine substitution in the linker region instead of that at the Zn(II) binding site can also sense and transmit the Zn(II) signal as well as wild type *P*. *aeruginosa*.

### The Zn(II) binding experiments of wild type and mutant CzcS SD

The chromogenic indicator 4-(2-Pyridylazo)resorcinol (PAR) is reported to form both 1:1 and 2:1 complexes with Zn(II) with stepwise affinity constants of 7.7×10^6^ and 5.0×10^5^ M^-1^, respectively (at pH 7.4, 0.15 M KCl, 22°C) [26, 27]. It has been widely used to determine the dissociation equilibrium constants of protein-Zn(II) complex in the range of nanomolar to picomolar [28, 29]. With the addition of Zn(II) to the PAR solution, the formative PAR-Zn and PAR_2_-Zn complex will cause an intense absorbance at 500 nm [29]. The absorption bands of PAR and Zn(II) complex at 500 nm are reduced by the addition of wild type and mutant CzcS SD (CzcS SD H55C, CzcS SD D60C, and CzcS SD L38C H55A) (**S5 Fig**). It indicates that the wild type CzcS SD and aforementioned mutants have the ability to compete with PAR for binding Zn(II). The representative titration spectrums are displayed in **S6 Fig** for the PAR with Zn (II) under the competition of wild type and mutant CzcS SD. The titration data at 500 nm and fitting binding isotherms of which are inserted in corresponding titration spectrums (**S6 Fig)**. The dissociation constants determined by Dynafit software [30] for Zn(II) with wild type CzcS SD, CzcS SD H55C, CzcS SD D60C, and CzcS SD L38C H55A are 1.7 (±0.2)×10^−6^ M, 8.5 (±0.4)×10^−7^ M, 5.7 (±0.3)×10^−8^ M, and 9.4 (±0.8)×10^−9^ M_,_ respectively. By using one-site fitting model, the coefficient of variation (CV) for each equilibrium binding experiments is approximately 10% regardless of whether the wild type or mutant CzcS SD is monitored (**S1 Table**). It indicates that the model for data fitting provides a good description of the available data.

### The Zn(II) induced dimerization of CzcS SD

The Zn(II) induced dimerization of CzcS SD was analyzed by the chemical crosslinking experiments (**S7A Fig**) with bis[sulfosuccinimidyl] suberate (BS^3^) as the primary amine reactive crosslinker [31–33]. Under denatured electrophoresis conditions, the CzcS SD primarily migrates at the position with the molecular weight of monomer, and negligible proportion of dimer is observed when it is treated by excess BS^3^ crosslinker (**S7A Fig**). It means that the CzcS SD without Zn(II) is mainly existed as monomer in solution that leads the poor efficiency of intermolecular crosslinking reaction. The efficiency of intermolecular crosslinking is significantly increased when Zn(II) is loaded into CzcS SD (**S7A Fig**). With the Zn(II) binding at dimer interface (**Fig 2A**), the dimer produced by the intermolecular crosslinking reaction is analyzed to be as high as 33 (±5) % of the original sample by using ImageJ analysis [34].

Other divalent cations such as Mg(II), Co(II), and Mn(II) are performed in the chemical crosslinking experiments, too. None of these divalent cations can induce crosslinked dimerization of wild type CzcS SD, which shows that Zn(II) is specific for this crosslinking (**S7A Fig**). However, the Co(II) show its ability in inducing the crosslinked dimerization of mutant CzcS SD D60C (**S7B Fig**). The proportion of dimer induced by Co(II) is less than that of Zn(II) (**S7C Fig**). This may be caused by the different coordination geometry preferred by Co(II) and Zn(II). As the data from Protein Data Bank (PDB) and Cambridge Structural Database (CSD), the octahedral geometry is preferred by Co(II) coordination and tetrahedral geometry is more preferred by Zn(II) coordination.

### Proline substitution of residues along the H1 and H1’ α-helices

**Fig 5:**
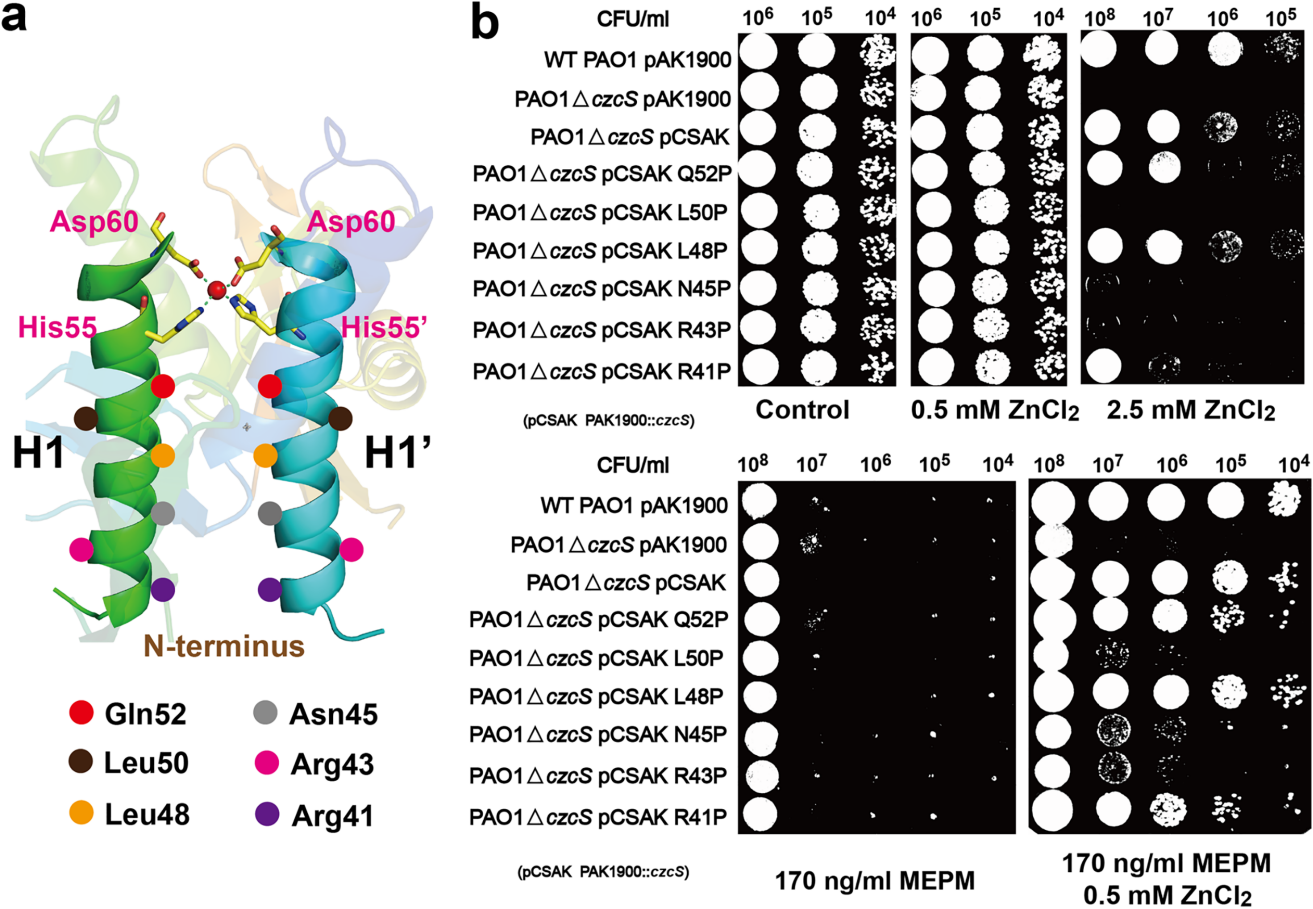
Proline substitution of residues along the H1 and H1’ α-helices. (a) The schematic diagram showing the locations of the residues involved in the proline substitutional experiments. The positions of Gln52, Leu50, Leu48, Asn45, Arg43, and Arg41 on the H1 and H1’ α-helices are labelled with circles in different colors. (b) Metal and antibiotic tolerance plate assay of single proline substitutions. Wild type *P*. *aeruginosa* and its derivative strains are examined on the LB plates that contain Zn(II) and MEPM antibiotic as follows: wild type *P*. *aeruginosa* with the empty pAK1900 plasmid as the control (WT PAO1 pAK1900), *czcS* deficient *P*. *aeruginosa* with empty pAK1900 (PAO1△*czcS* pAK1900), *czcS*-deficient *P*. *aeruginosa* with wild type *czcS* encoded on pAK1900 (PAO1△*czcS* pCSAK), *czcS*-deficient *P*. *aeruginosa* complemented with *czcS* mutants in pAK1900 (PAO1△*czcS* pCSAK Q52P, PAO1△*czcS* pCSAK L50P, PAO1△*czcS* pCSAK L48P, PAO1△*czcS* pCSAK N45P, PAO1△*czcS* pCSAK R43P, and PAO1△*czcS* pCSAK R41P).

It is well known that proline residue will distort the regular structure of helices by introducing a kink between the segments adjacent to it [35–37]. In this study, we want to characterize whether the distortion of H1 and H1’ α-helices has any influence on the function of HK CzcS by the introduction of proline residue. Besides the residues adjacent to the C-terminal Zn(II) binding site, other residues along the H1 and H1’ α-helices are chosen to do the proline substitutions. The residues Gln52, Leu50, Leu48, Asn45, Arg43, and Arg41 arranged with almost equal interval are replaced by proline residues **(Fig 5A)**. All these single proline substitutional mutants grow well in the low concentration of Zn(II) (0.5 mM) and respond to MEPM equivalently to that of wild type *P*. *aeruginosa*. However, most of the mutants (L50P, N45P, R43P, and R41P) display varying degrees of impairments in their resistance to higher concentration of Zn(II) (2.5 mM) and the Zn(II)-inducible cross-resistance to MEPM **(Fig 5B)**. Unlike the proline substitutions, other non-conserved mutations **(S2 Table)** of these residues (Arg41, Arg43, and Arg45) do not profoundly affect the signaling response. We also find that some proline substitutions still play full functions in Zn(II) induced metal detoxification and MEPM antibiotic resistance, such as Q52P and L48P **(Fig 5B)**. Further, we also do some double proline substitutions along the H1 and H1’ α-helices (**S8 Fig**). All the double mutants (R41P L48P, R43P L48P, N45P L48P, R41P N45P, R43P N45P, and R41P R43P) totally lose the functions in Zn(II) induced metal detoxification and MEPM antibiotic resistance.

## Discussion

TCSs are frequently used by bacteria to adapt to the dynamic environments by coping with external stimuli [1–5]. Since their first discovery approximately 25 years ago, TCSs have been extensively identified in microorganisms. Although more and more crystal structures of HKs have been determined in the last few years [38, 39], the crystallographic analyses of Zn(II)-binding senor domains of HKs have not been reported to date. What’s more, the mechanisms by which extracellular stimuli are transduced from the sensor domain to the intracellular kinase domains are one of the least understood aspects of TCS response [38]. With growing concerns about co-regulation between heavy metals and antibiotic resistance [13], the CzcR-CzcS TCS of the pathological bacterium *P*. *aeruginosa* is an excellent candidate to study on. Here, we present a high-resolution structure of the CzcS SD in complex with its cognate ligand, Zn(II). The characterized Zn(II)-bound CzcS SD is demonstrated as a functional dimer with its central parallel bundle formed by the H1 and H1’ α-helices (**Fig 2A**). The subunit of the CzcS SD reveals characteristic PDC folds with N-terminal helix-loop-helix domain that leads into the central five anti-parallel β-sheet scaffold (**Fig 1C**). The N-terminal and C-terminal ends of the CzcS SD are orient in parallel, which allows communication of the connected transmembrane segments with the structural character of dimeric four-helical bundles [40].

The topologically similar PDC members, such as PhoQ, DcuS, CitA, DctB, and CzcS sensors, exhibit an enormous sequence variability (**S2 Fig**). Within the common structural arrangement, the sequence varieties enable them to detect diverse stimuli. Most of the characterized PDC sensor domains (CitA, DcuS, and DctB) detect their cognate ligands by the internal cavity that is formed by the central conserved β-sheets (**Fig 1C**) [20, 22]. Differently, the extracellular sensor domain of CzcS utilizes the residues from the symmetrical N-terminal α-helices to coordinate with Zn(II) in a tetrahedral coordination geometry. This constitutes a special class of Zn(II) binding sites that form at the dimer interface in the biochemical Zn(II) sites [24]. The recently reported metal-ion sensor CusS binds the effector at the dimer interface as well. However, the CusS SD interacts with the Ag(I) by using the N-terminal and C-terminal α-helices separately from different monomers. In the crystal structure of CzcS-Zn, the average distance is approximately 14.9 Å between the H1 and H1’ α-helices **(Fig 2A)**. In the absence of outer-shell constraints **(S9A Fig)**, the H1 and H1’ α-helices are flexible in rearranging the structural orientation. Their minor reorientation will initiate a large readjustment that affects the Zn(II) binding site **(S9B Fig)**. These structural features enable the rapid regulation of the active site for Zn(II) binding or releasing. It is similar to that of the Zn(II) binding site confined between TM2 and TM5 in the Zn(II) transporter YiiP [41].

In the CzcS SD, the coordination environment is symmetrical with the His55, Asp60, His55’, and Asp60’ residues from the H1 and H1’ α-helices, respectively **(Fig 2A)**. This class of Zn(II) ligands that comprise the His and Asp residues is rare for the reported biochemical Zn(II) sites [42]. It makes the CzcS SD bind to Zn(II) with an affinity of 1.7 (±0.2)×10^−6^ M. The RT-PCR analysis also indicates that the expression levels of *czcS*, *czcR*, *czcC* as well as *oprD* have obviously up-regulation or down-regulation when the *P*. *aeruginosa* is stimulated by Zn(II) at a micromole level **(S10 Fig)**. When the Zn(II) ligands of HK CzcS are substituted by cysteine residues, the mutants H55C and D60C can respond to Zn(II) stimulus as well and bind Zn(II) with higher affinities than that of the wild type construct *in vitro*. For the D60C mutant, a classic Cys_2_His_2_ zinc finger configuration with a tetrahedral coordination geometry **(S11A Fig)** can be properly formed by the residues Cys60, Cys60’, His55, His55’ with Zn(II) [43]. The similar coordination geometry to that of the wild type HK CzcS causes the D60C mutant to display equivalent activities in sensing and regulating Zn(II) signal (**Fig 2C**). We speculate that a linear coordination geometry may be formed by Cys55 and Cys55’ with Zn(II) in the H55C mutant, which is similar to the configuration formed on the dimer interface of the colicin E3 immunity protein **(S11B Fig)** [42, 44]. The H55C mutant strain maintains the ability to respond Zn(II) stimulus as well (**Fig 2C**).

The biologically relevant dimer is observed in the crystal structure of CzcS-Zn complex with Zn(II) binding at the dimer interface (**Fig 1B**). We ever made great efforts but failed in crystallizing the CzcS SD in the absence of Zn(II). The difficulties in crystallization may be predominantly caused by the high flexibility of the CzcS SD especially the swing of N-terminal α-helices. In the absence of Zn(II), the CzcS SD is exited as monomer in solution. When it binds to Zn(II), the CzcS SD transforms from monomer to dimer and seems to be more conformational stable with the H1 and H1’ α-helices confined by Zn(II) coordination. Along with *in vivo* biological evidences, the Zn(II) induced dimerization of the CzcS SD is supposed to be physically important for signal regulation.

This speculation is also confirmed by the different effects of Co(II) on the regulation of antibiotic resistance between wild type and mutant D60C strain (**Fig 3**). The D60C strain turns to be a Co(II)-responsive regulator that shows Co(II)-inducible resistance to MEPM. *In vitro*, the crosslinked dimerization of sensor domain induced by Co(II) is also observed for the D60C mutant. These experiments again indicate that the association of H1 and H1’ α-helices is necessary for the activity of HK CzcS. What’s more, the association state of H1 and H1’ α-helices maintains till to the linker region when HK CzcS is in the activated form. In the case that the original Zn(II) binding site is destroyed (mutant H55A), a cysteine substitution in the linker region (mutant L38C H55A) can bind to Zn(II) with high affinity *in vitro* and strongly respond to the Zn(II) stimulus *in vivo* as well (**Fig 4**).

The rigid structural features of H1 and H1’ α-helices are the pivotal guarantee for the signal transduction, and this is verified by the proline substitutional experiments. Proline residues are known to distort the structure of helices [45, 46]. Visual inspection of some helices with a proline residue demonstrates a range of helix distortion (**S12 Fig**). Obvious kink angle is observed in some proline-containing α-helices (**S12A Fig**), which may happen to the H1 and H1’ α-helices in the mutant strains L50P, N45P, R43P, and R41P. This kind of distortion of H1 and H1’ α-helices makes the mutant strains (L50P, N45P, R43P, and R41P) seriously impair in the functions of Zn(II) induced metal detoxification and antibiotic resistance. While, there also exist some proline-containing α-helices that are approximately straight (**S12B Fig**). It may be the reason that why the growing status of mutants Q52P and L48P is not influenced by the introduction of proline residue on H1 and H1’ α-helices. In addition, the H1 and H1’ α-helices can’t resist the double proline substitutions, which lead serious impairments in the abilities of responding to Zn(II) signal. Thus, it’s important to keep the conformation of H1 and H1’ α-helices in the signal transduction.

**Fig 6:**
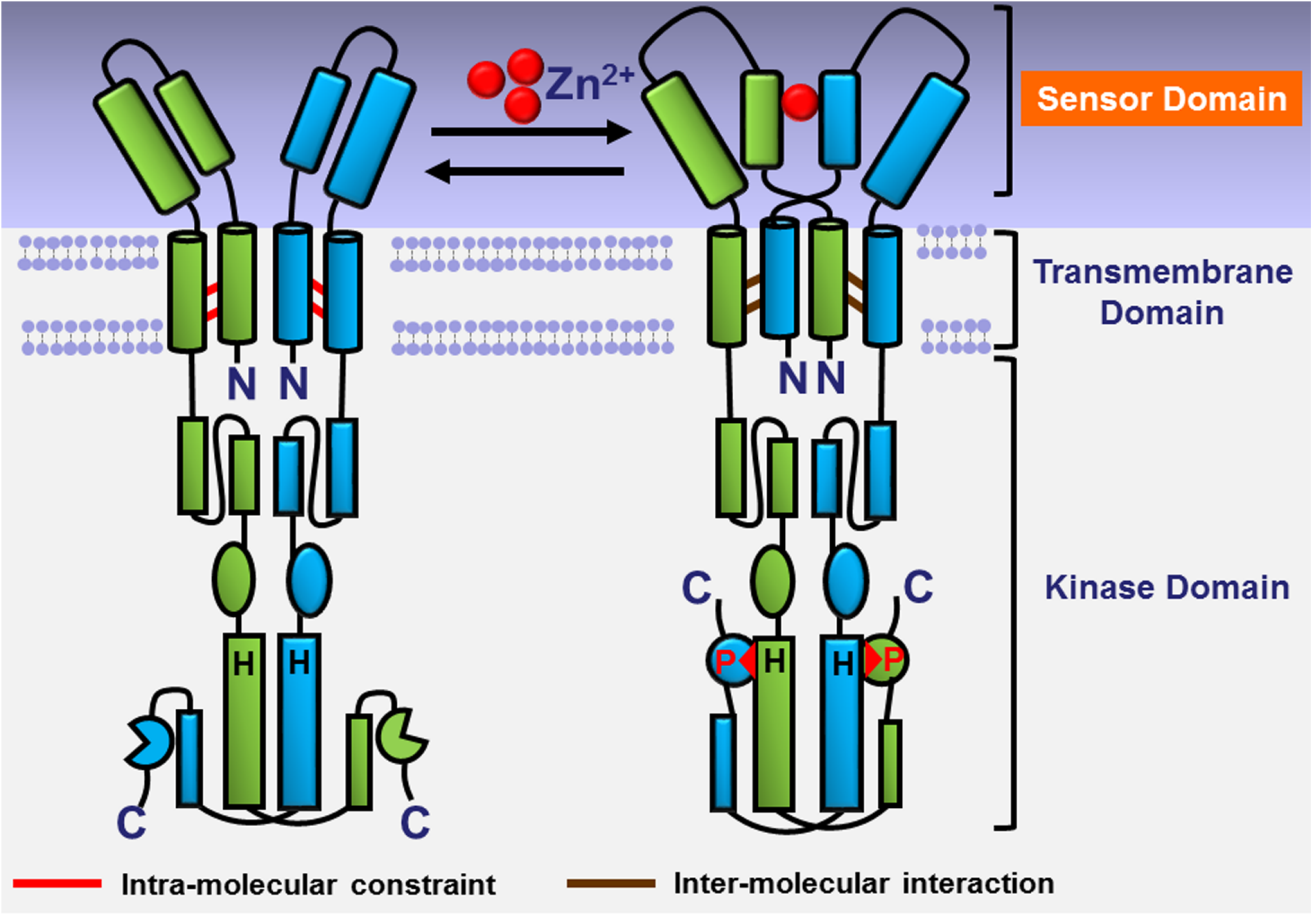
Proposed molecular mechanism of Zn(II) signal transduction in HK CzcS. The two monomers in the CzcS functional dimer are shown in green and in cyan. When the Zn(II) binds to CzcS, the sensor domain will turn from monomer to dimer with the Zn(II) binding at the symmetrical N-terminal α-helices. The dimerization of sensor domain will drive the interactional rearrangement within the dimeric four-helical bundles in the transmembrane domain [47], and autophosphorylation is activated at the conserved histidine residues in the cytoplasmic kinase domain [48–51].

As above described, the H1 an H1’ α-helices are the key factors in the activity of HK CzcS. They interact with the Zn(II) and keep the Zn(II)-induced association state till to the linker region, which are physiologically important for extracellular signal sensing and transduction across the transmembrane helices to the cytoplasmic kinase (**Fig 6**). By other group research, the rearranged helical interactions are discovered within the dimeric four-helical bundles in the transmembrane domain when HK CzcS is activated [47]. There is a transition from intramolecular- to intermolecular-crosslinking within the transmembrane helices (**Fig 6**) [47]. We speculate it’s the association of H1 an H1’ α-helices that leads the structural rearrangements in the sensor domain, which will drive the interactional displacements of the helix bundles in the transmembrane domain. The aforementioned quaternary structural changes within the homodimer ultimately lead the *trans* autophosphorylation in cytoplasmic kinase domain on the conserved histidine residues (**Fig 6**) [48–51]. The promising model (**Fig 6**) presented here provides preliminary insights into the molecular mechanism of Zn(II) signal sensing and transduction by HK CzcS. It gives an implication for understanding the Zn(II) induced metal detoxification and antibiotic resistance in CzcR-CzcS TCS of *P*. *aeruginosa*. However, besides the structural information of extracellular sensor domain provided in this study, the structural characterizations of transmembrane domain and cytoplasmic kinase domain have not been reported to date for HK CzcS. Thus, further investigations are still needed to precisely characterize how the signal in the sensor domains results in the interactional rearrangement of the transmembrane helices and modulates the autophosphorylation events in the cytoplasmic kinase domains.

## Materials and methods

### Construction, expression, and purification of the wild type and mutant CzcS SD

The DNA fragment of the CzcS SD (amino acids 40–166 in CzcS protein) was amplified from *P*. *aeruginosa* genomic DNA and cloned into the NheI and HindIII sites of the pET-28a vector (named pCSET). *E*. *coli* BL21 (DE3) cells transformed with the construct were cultivated in the LB medium supplemented with 30 μg/ml of kanamycin. The cells were cultivated at 37°C with constantly shaking at 250 rpm following a 1:100 inoculation from an overnight culture. Expression was induced with 0.5 mM IPTG when the culture reached an optical density of OD _600_ ≈ 0.6. The induced cells were grown for 4 h at 30°C, and subsequent steps were performed at 4°C. Cells expressing the CzcS SD with an N-terminal His_6_-Tag were harvested by centrifugation and lysed by sonication on ice in 15 ml of lysis buffer (10 mM Tris-HCl, pH 7.4, 100 mM NaCl, 0.1 mM PMSF, 10% glycerol, and 1 μl DNaseI). The supernatant was obtained by centrifugation at 12,000 rpm for 15 min and loaded onto a 5-ml Ni-NTA column that was pre-equilibrated with 2–3 column volumes of buffer A (10 mM Tris-HCl, pH 7.4, 100 mM NaCl, and 25 mM imidazole). The fusion protein was eluted in a linear gradient with the concentration of imidazole ranging from 75 mM to 500 mM in buffer A. The N-terminal His_6_-tag was removed by digesting the fusion protein with a protease overnight. The His_6_-tag-cleaved protein was treated with 0.5 mM EDTA and purified on an 8-ml MonoQ anion-exchange column which was equilibrated with buffer B (10 mM Tris-HCl, pH 7.4, and 50 mM NaCl). The protein was eluted from the MomoQ column with 100 mM NaCl in buffer B. The purified CzcS SD were identified by SDS-PAGE and used in the following experiments.

The CzcS SD mutants (CzcS SD H55C and CzcS SD D60C) were obtained by using site-directed mutagenesis technology performed on the pCSET plasmid. The CzcS SD L38C H55A mutant (amino acids 27–175 in CzcS protein) was constructed in the same method as pCSET plasmid followed by site-directed mutagenesis of sites Leu38 and His55. All the CzcS SD mutants were expressed and purified in the same procedures as wild type CzcS SD.

### Crystallization, data collection, and structure determination

To crystallize the CzcS-Zn, the CzcS SD with the concentration of 3–4 mg/ml was mixed with an equimolar amount of ZnSO_4_ in the buffer containing 10 mM Tris-HCl, pH 8.5, and 100 mM NaCl. The complex crystals were grown at 16°C by the sitting-drop vapor-diffusion against the reservoir buffer containing 0.2 M (NH_4_)_2_SO_4_, 0.1 M Bis-Tris pH5.5, and 25% w/v polyethylene glycol 3350. The irregular cuboid crystals came out after two days and continued to grow until reaching a suitable size for X-ray diffraction studies. The crystals were briefly soaked in a cryoprotectant containing 0.2 M (NH_4_)_2_SO_4_, 0.1 M Bis-Tris pH5.5, 25% w/v polyethylene glycol 3350, and 8% glycerol prior to flash-frozen in liquid nitrogen.

The diffraction datasets at the zinc K-edge were collected from single crystals at BL17U beamlines at the Shanghai Synchrotron Radiation Facility [52]. The X-ray diffraction datasets were integrated and scaled with the HKL2000 package software. The initial phase for automated model building was solved by zinc single-wavelength anomalous dispersion using the Phenix software [53]. Iterative rounds of refinement were performed using the Phenix software, which were followed by manual alterations using the WinCoot software [54]. Refinement was conducted until no significant improvements were achieved. All structural models for the current study were generated with the PyMOL software [55]. The data collection and refinement statistics are listed in **Table 1**. The atomic coordinates and structural factors for CzcS-Zn have been deposited into the Protein Data Bank with accession code 5GPO.

### Construction of the *czcS*-deficient strain of *P*. *aeruginosa*

The *czcS*-deficient strain of *P*. *aeruginosa* was constructed with a homologous recombination assay [56]. A 2025-bp PCR fragment corresponding to the first 8 bp of the *czcS* gene was amplified from *P*. *aeruginosa* genomic DNA with the primers I and II which contain an EcoRI and an XbaI restriction sites, respectively. Another 1892-bp PCR fragment that contains the 3’-end of the *czcS* gene was amplified from *P*. *aeruginosa* genomic DNA with primers III and IV which contain an XbaI and a HindIII restriction sites, respectively. The intervening gentamicin resistance cassette was amplified from the pPS858 plasmid with the XbaI restriction site both at 5’- and 3’-end. The aforementioned three DNA fragments were ligated into an EcoRI /HindIII-cleaved pEX18AP plasmid. The constructed plasmid was transformed into the *P*. *aeruginosa* competent cells by electroporation [56]. The successfully homologous recombinants were screened on the LB medium containing 30 ug/ml gentamycin. The *czcS*-deficient strain was further identified by PCR and DNA sequencing. The oligonucleotides used to construct the *czcS*-deficient strain are listed in **S3 Table**.

### Construction of complementary plasmid and its mutants

The *czcS* operon and its encoding gene were amplified from *P*. *aeruginosa* genomic DNA. They were ligated by overlap PCR and cloned into the HindIII/BamHI restriction sites of pAK1900. The complementary plasmid pCSAK was verified by DNA sequencing and used to construct the mutants. Site-directed mutagenesis of CzcS was performed on the yielding pCSAK plasmid using the Quik Change site-directed mutagenesis kit (Agilent Technologies). All the variants were verified by DNA sequencing. The oligonucleotides used for the site-directed mutagenesis plasmids are listed in **S3 Table**.

### Metal and antibiotic tolerance plate assay

The pCSAK plasmid was transformed into the *czcS*-deficient strain by chemical transformation to supply as the complementary strain. The empty pAK1900 plasmid was transformed into the wild type *P*. *aeruginosa* and *czcS*-deficient strain to supply as the positive control and negative control, respectively. All the variants were separately transformed into the *czcS*-deficient strain. The aforementioned strains were cultivated in LB mediums supplemented with 150 μg/ml carbenicillin. They were grown overnight at 37°C with constantly shaking at 250 rpm/min. Fresh LB mediums containing 150 μg/ml carbenicillin were inoculated with the overnight cultures at the proportion 1:100 and grown at 37°C until the density of OD _600_ arrived 1.0. The cultures then underwent ten-fold serial dilutions with five gradients and were further seeded onto the LB plates with varying concentrations of Zn(II), Co(II) or MEPM. The plates were incubated at 37°C for 16 h before observation.

### Competitive Zn(II) binding experiments with 4-(2-Pyridylazo)resorcinol (PAR)

The chromogenic chelating agent PAR was selected as the competitor in the spectrometric determinations of binding affinity of Zn(II) with wild type and mutant CzcS SD (CzcS SD H55C, CzcS SD D60C, and CzcS SD L38C H55A). All Zn(II) binding experiments were performed under photophobic condition at 22°C in the buffer containing 10 mM Tris-HCl, pH 7.4, 150 mM NaCl, and 0.5 mM TCEP (for the mutants CzcS SD H55C, CzcS SD D60C, and CzcS SD L38C H55A only). A known concentration of PAR solution (36 uM) was mixed with the purified protein (50 uM-200 uM). The mixtures were divided into equal volumes followed by loading consistent volumes of ZnCl_2_ with increasing concentrations (in the range of 2 uM-42 uM). The UV-visible spectrum were recorded in the range of 200 nm to 700 nm until the reaction systems achieved competitive equilibrium. The titration data at 500 nm were fit with Dynafit software [30] by using one-site model to obtain the apparent dissociation constants of Zn(II) with wild type and mutant CzcS SD.

### Intermolecular chemical crosslinking with bis[sulfosuccinimidyl]) suberate (BS^3^) as the crosslinker

The CzcS SD was purified in the same procedures as described above in the buffer containing 10 mM HEPES, pH7.4, 100 mM NaCl. The primary amine reactive crosslinker BS^3^ was stored in DMSO at 100 mM and diluted to 1 mM in 20mM HEPES (pH7.4) immediately before use. A 50-fold molar excess of BS3 crosslinker was loaded into the CzcS SD and CzcS-Zn samples with a final concentration of 1 mM. The reaction systems were incubated at room temperature for 30 minutes and quenched with 50 mM Tris-HCl, pH 7.4. The quenching reaction was incubated at room temperature for 15 minutes. The CzcS SD, CzcS-Zn, and the products of the crosslinking reactions were analyzed by 14% SDS-PAGE and quantified by ImageJ [34]. The crosslinking experiments of other divalent cations, such as Mg(II), Mn(II), or Co(II), were performed in the same procedure.

### Quantitative real-time RT-PCR

The quantitative real-time RT-PCR with the *rpsl* gene as the reference was performed to monitor the expression changes of *czcS*, *czcR*, *czcC* and *oprD* genes when *P*. *aeruginosa* is stimulated by the Zn(II) at a micromolar level. The total RNA was extracted by traditional phenol-chloroform method and reverse transcribed by iScript cDNA Synthesis Kit (Bio-Rad). The cDNA samples were diluted for different folds and used as the templates in the PCR experiments. The real-time RT-PCR was performed on the Bio-Rad CFX96 equipment using the Ssofast SYBR Green Supermix (Bio-Rad). The experiments were performed at least three independent times with average results shown. The primer sequences used for real-time RT-PCR are designed using the Primer3 program and listed in **S3 Table**.

## Supporting information

S1 Fig The two molecules in the structure of CzcS-Zn. (a) Conformations of the two CzcS molecules observed in the structure of CzcS-Zn. The two CzcS molecules are shown as cartoons in gray and green. The Zn(II) ions are shown as spheres in red. (b) The structural superimposition of the two molecules in cartoon representation. Secondary structural elements are labeled on their corresponding positions in black. The superimposition performed with the Pymol software yields an r.m.s.d. of 0.8 Å over 104 pairs of Cα atoms.
(TIF)

S2 Fig Structure-based sequence alignments of the CzcS SD with other PDC members. The secondary structural elements of the CzcS SD are labeled above the alignments with α-helices (amaranthine panes) and β-strands (blue arrows). The secondary structural elements of the other proteins are indicated by corresponding colors (amaranthine for α-helices and blue for β-strands) within the protein sequences. Organism names are abbreviated as (Pa for *Pseudomonas aeruginosa*, Ec for *Escherichia coli*, and Kp for *Klebsiella pneumonia*).
(TIF)

S3 Fig The coordination of the functionally irrelevant Zn(II) ion caused by the crystallographic packing. The three CzcS molecules (A, B, and C) are shown as cartoon representations and indicated in gray, cyan, and magenta, respectively. The Zn(II) ion is coordinated in the tetrahedral geometry with His72 and Asp76 from molecule A, Asp62 form molecule B, and His72 from molecule C.
(TIF)

S4 Fig Identification of the Zn(II) ligands of the HK CzcS by plate assay. Wild type *P*. *aeruginosa* and its derivative strains are examined on LB plates that contain Zn(II) ions: wild type *P*. *aeruginosa* with the empty pAK1900 plasmid as the control (WT PAO1 pAK1900), *czcS*-deficient *P*. *aeruginosa* with empty pAK1900 (PAO1△*czcS* pAK1900), *czcS-*deficient *P*. *aeruginosa* with wild type *czcS* encoded on pAK1900 (PAO1△*czcS* pCSAK), and *czcS*-deficient *P*. *aeruginosa* complemented with *czcS* mutants in pAK1900 (PAO1△*czcS*H55A D60A and PAO1△*czcS* H72A D76A).
(TIF)

S5 Fig Titration of PAR and Zn(II) complex by the wild type and mutant CzcS SD. The control spectrum was recorded in the presence of 36 uM PAR and 18 uM ZnCl_2_. The absorption bands of 36 uM PAR and 18 uM Zn(II) at 500 nm were reduced by the addition of wild type and mutant CzcS SD (CzcS SD H55C, CzcS SD D60C, and CzcS SD L38C H55A).
(TIF)

S6 Fig Competitive Zn(II) titration experiments of wild type and mutant CzcS SD with PAR. The representative titration UV spectrum of PAR (36uM) with the increasing Zn(II) (2 uM-42 uM) was recorded in the presence of (a) 68 uM CzcS SD, (b) 105 uM CzcS SD H55C, (c) 60 uM CzcS SD D60C, and (d) 72 uM CzcS SD L38C H55A competition in the range of 200 nm to 700 nm. The titration data at 500 nm and fitting binding isotherm by using Dynafit software [30] were inserted in corresponding titration UV spectrum.
(TIF)

S7 Fig The intermolecular crosslinking of wild type and mutant CzcS SD by using BS3 as the crosslinker. The monomer and dimer of wild type and mutant CzcS SD are indicated with ● and ●●, respectively. (a) The Zn(II) induced crosslinked dimerization of wild type CzcS SD. Other divalent cations such as Mg(II), Co(II), and Mn(II) in the experiments are supplied as negative control. (b) The Co(II) induced crosslinked dimerization of CzcS SD D60C mutant. (c) The Zn(II) induced crosslinked dimerization of CzcS SD D60C mutant.
(TIF)

S8 Fig Metal and antibiotic tolerance plate assay of double proline substitutions. Wild type *P*. *aeruginosa* and its derivative strains are examined on the LB plates that contain Zn(II) and MEPM antibiotic as follows: wild type *P*. *aeruginosa* with the empty pAK1900 plasmid as the control (WT PAO1 pAK1900), *czcS* deficient *P*. *aeruginosa* with empty pAK1900 (PAO1△*czcS* pAK1900), *czcS*-deficient *P*. *aeruginosa* with wild type *czcS* encoded on pAK1900 (PAO1△*czcS* pCSAK), *czcS*-deficient *P*. *aeruginosa* complemented with *czcS* mutants in pAK1900 (PAO1△*czcS* pCSAK R41P L48P, PAO1△*czcS* pCSAK R43P L48P, PAO1△*czcS* pCSAK N45P L48P, PAO1△*czcS* pCSAK R41P N45P, PAO1△*czcS* pCSAK R43P N45P, and PAO1△*czcS* pCSAK R41P R43P).
(TIF)

S9 Fig The outer-shell environment and regulation of Zn(II) coordination geometry. (a)The outer-shell environment of the H1 and H1’ α-helices. Other than the water molecule-mediated hydrogen-bond, there are no direct interactions between the residues of the H1 and H1’ α-helices. (b) Regulation of Zn(II) coordination geometry. A close-up view of the Zn(II) binding site confined between the H1 and H1’ α-helices (shown in cylinders). The black arrows indicate the hypothesized movements of the H1 and H1’ α-helices.
(TIF)

S10 Fig Relative fold changes of the *czcS*, *czcR*, *czcC*, and *oprD* genes with *P*. *aeruginosa* induced by 10 uM Zn(II). The experiments are performed in duplicate with the average results and standard deviations shown.
(TIF)

S11 Fig The Zn(II) coordination geometry formed in the zinc finger protein and colicin E3 immunity protein. (a) In the D60C mutant, a tetrahedral coordination geometry analogy to the classic Cys_2_His_2_ zinc finger (PDB code: 1NCS) can be formed [43]. (b) In the H55C mutant, a linear coordination geometry can be formed between dithiolate and Zn(II), which is similar to that is formed on the dimer interface of the colicin E3 immunity protein from (PDB code: 3EIP) [44].
(TIF)

S12 Fig A selection of proline-containing α-helices [57–61]. The helix is shown as cartoon in slate on which proline residue is shown as stick in magenta. (a) The proline-containing α-helices with obvious kink angle. (b) The proline-containing α-helices with no distortion.
(TIF)

S1 Table The model parameters analyzed by DynaFit in determining the dissociation constants of wild type and mutant CzcS SD with Zn(II). (DOC)

S2 Table Non-conservative mutations of residues along the H1 and H1’ α-helices. (DOC)

S3 Table Oligonucleotides used in this study. (DOC)
