# Supplementary figures and images for: Structural basis of Zn(II) induced metal detoxification and antibiotic resistance by histidine kinase CzcS in Pseudomonas aeruginosa
Source: PLoS Pathog. 2017 Jul 21;13(7):e1006533. doi: 10.1371/journal.ppat.1006533 (PMC5540610; doi:10.1371/journal.ppat.1006533)

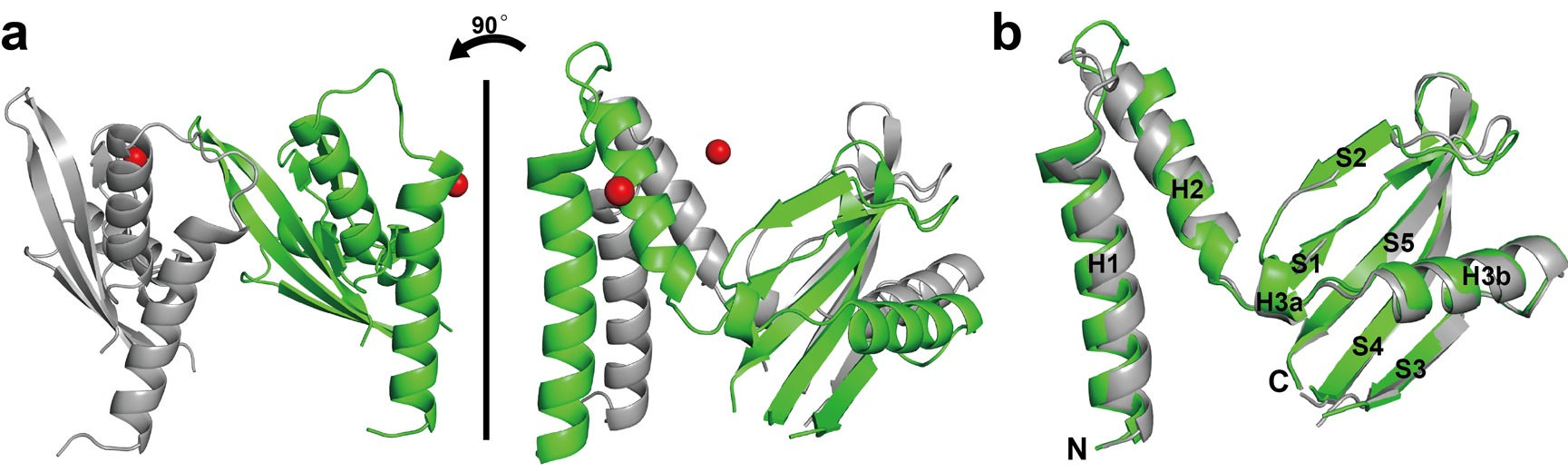

Supplement: S1 Fig — (a) Conformations of the two CzcS molecules observed in the structure of CzcS-Zn. The two CzcS molecules are shown as cartoons in gray and green. The Zn(II) ions are shown as spheres in red. (b) The structural superimposition of the two molecules in cartoon representation. Secondary structural elements are labeled on their corresponding positions in black. The superimposition performed with the Pymol software yields an r.m.s.d. of 0.8 Å over 104 pairs of Cα atoms. (TIF) [file ppat.1006533.s001.tif]

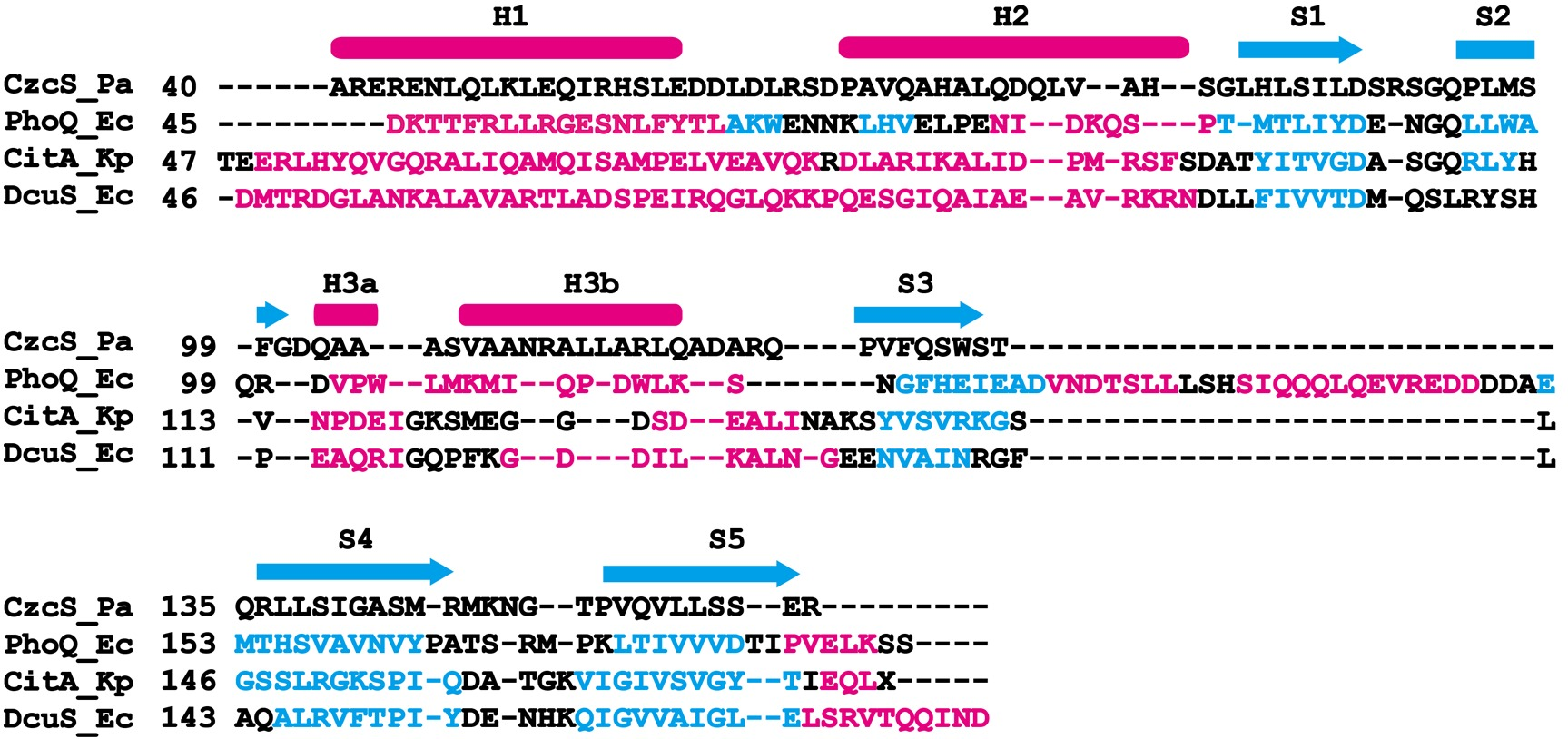

Supplement: S2 Fig — The secondary structural elements of the CzcS SD are labeled above the alignments with α-helices (amaranthine panes) and β-strands (blue arrows). The secondary structural elements of the other proteins are indicated by corresponding colors (amaranthine for α-helices and blue for β-strands) within the protein sequences. Organism names are abbreviated as (Pa for Pseudomonas aeruginosa, Ec for Escherichia coli, and Kp for Klebsiella pneumonia). (TIF) [file ppat.1006533.s002.tif]

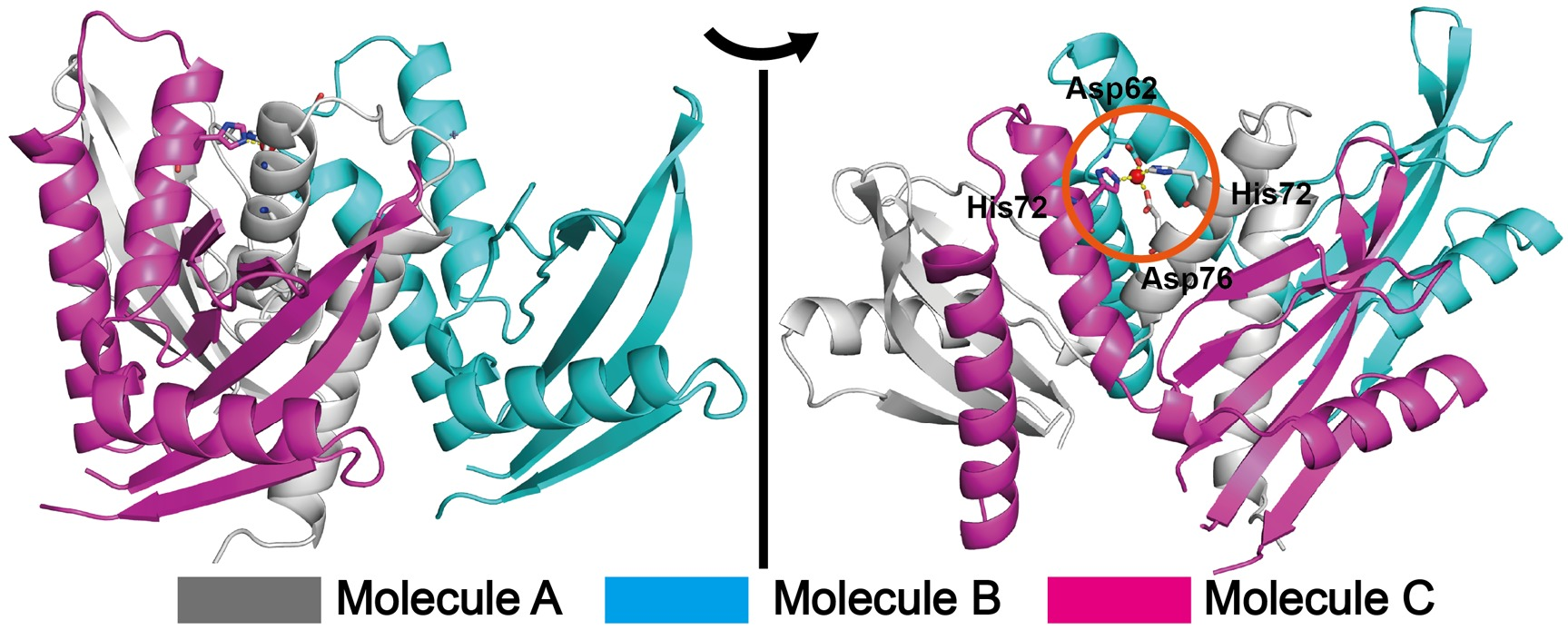

Supplement: S3 Fig — The three CzcS molecules (A, B, and C) are shown as cartoon representations and indicated in gray, cyan, and magenta, respectively. The Zn(II) ion is coordinated in the tetrahedral geometry with His72 and Asp76 from molecule A, Asp62 form molecule B, and His72 from molecule C. (TIF) [file ppat.1006533.s003.tif]

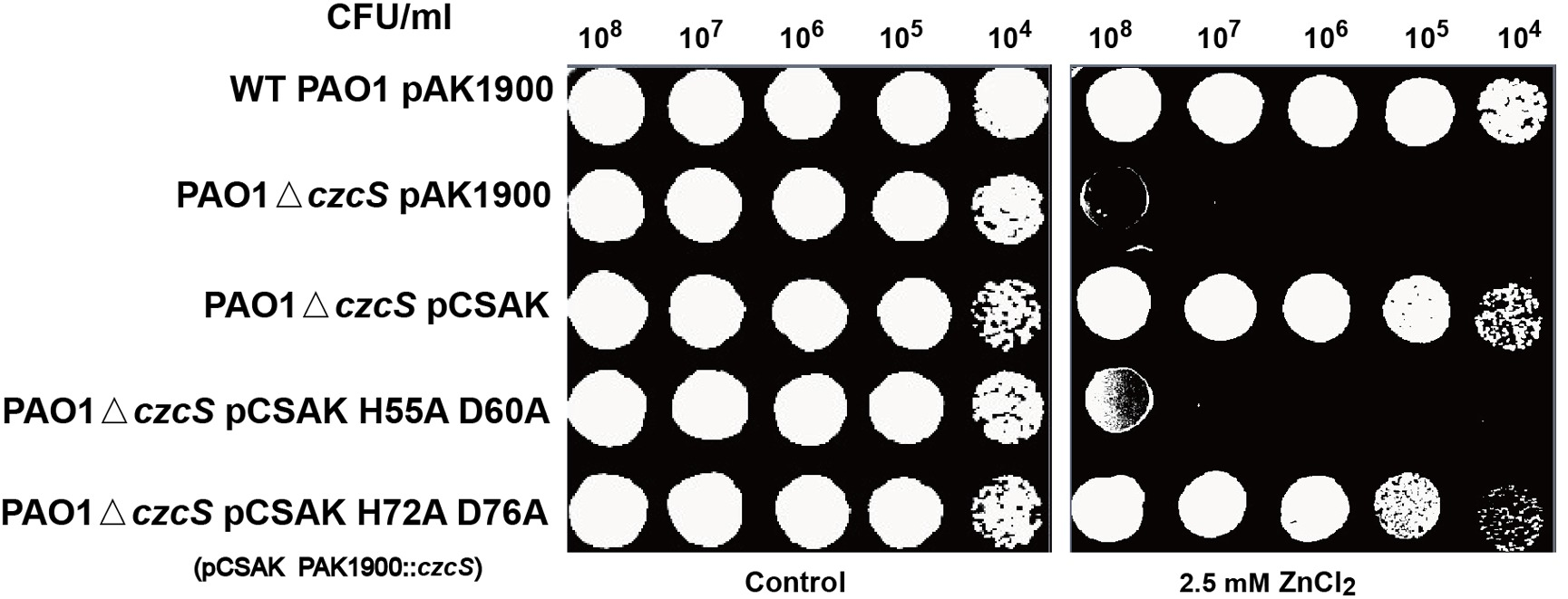

Supplement: S4 Fig — Wild type P. aeruginosa and its derivative strains are examined on LB plates that contain Zn(II) ions: wild type P. aeruginosa with the empty pAK1900 plasmid as the control (WT PAO1 pAK1900), czcS-deficient P. aeruginosa with empty pAK1900 (PAO1△czcS pAK1900), czcS-deficient P. aeruginosa with wild type czcS encoded on pAK1900 (PAO1△czcS pCSAK), and czcS-deficient P. aeruginosa complemented with czcS mutants in pAK1900 (PAO1△czcSH55A D60A and PAO1△czcS H72A D76A). (TIF) [file ppat.1006533.s004.tif]

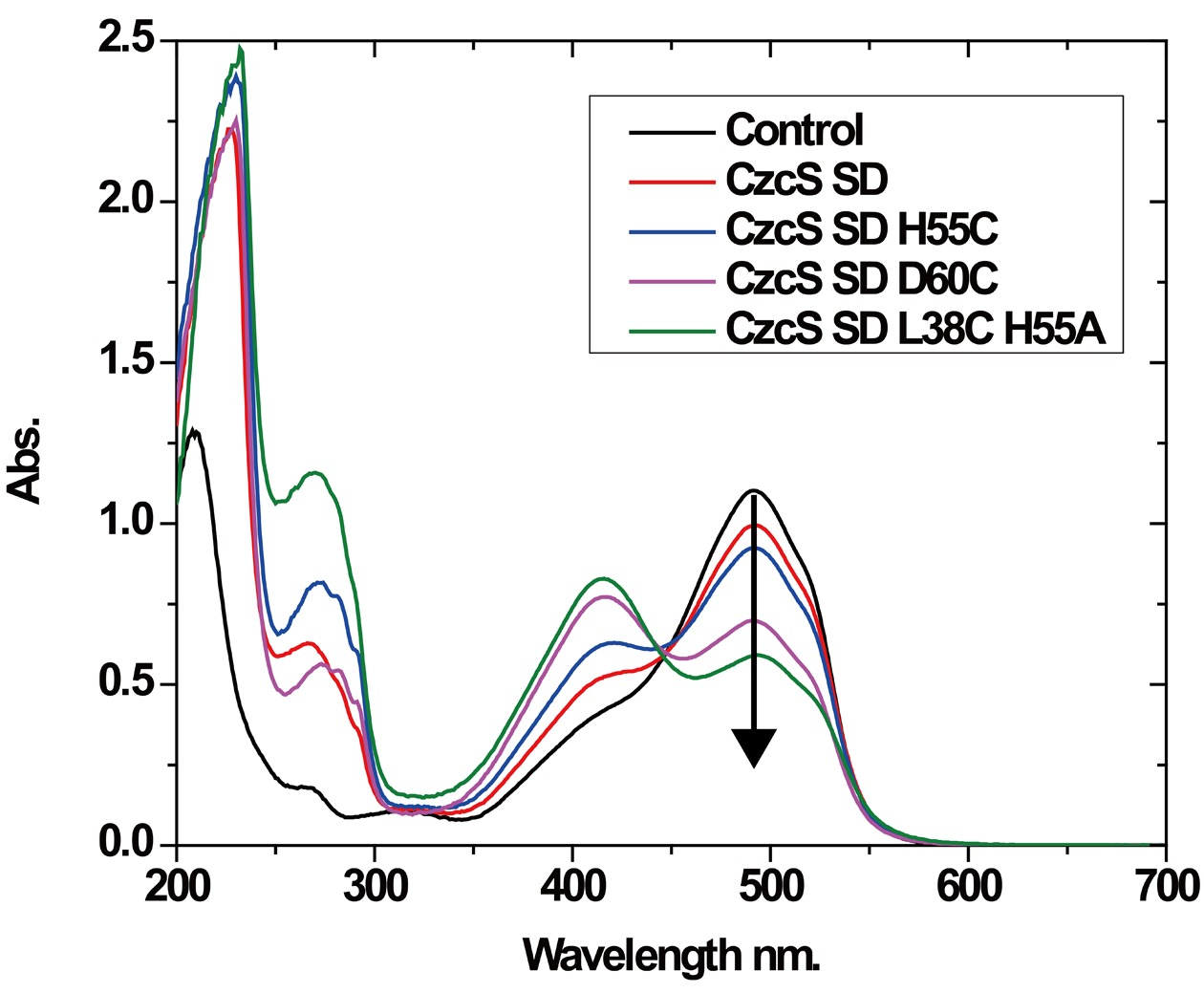

Supplement: S5 Fig — The control spectrum was recorded in the presence of 36 uM PAR and 18 uM ZnCl2. The absorption bands of 36 uM PAR and 18 uM Zn(II) at 500 nm were reduced by the addition of wild type and mutant CzcS SD (CzcS SD H55C, CzcS SD D60C, and CzcS SD L38C H55A). (TIF) [file ppat.1006533.s005.tif]

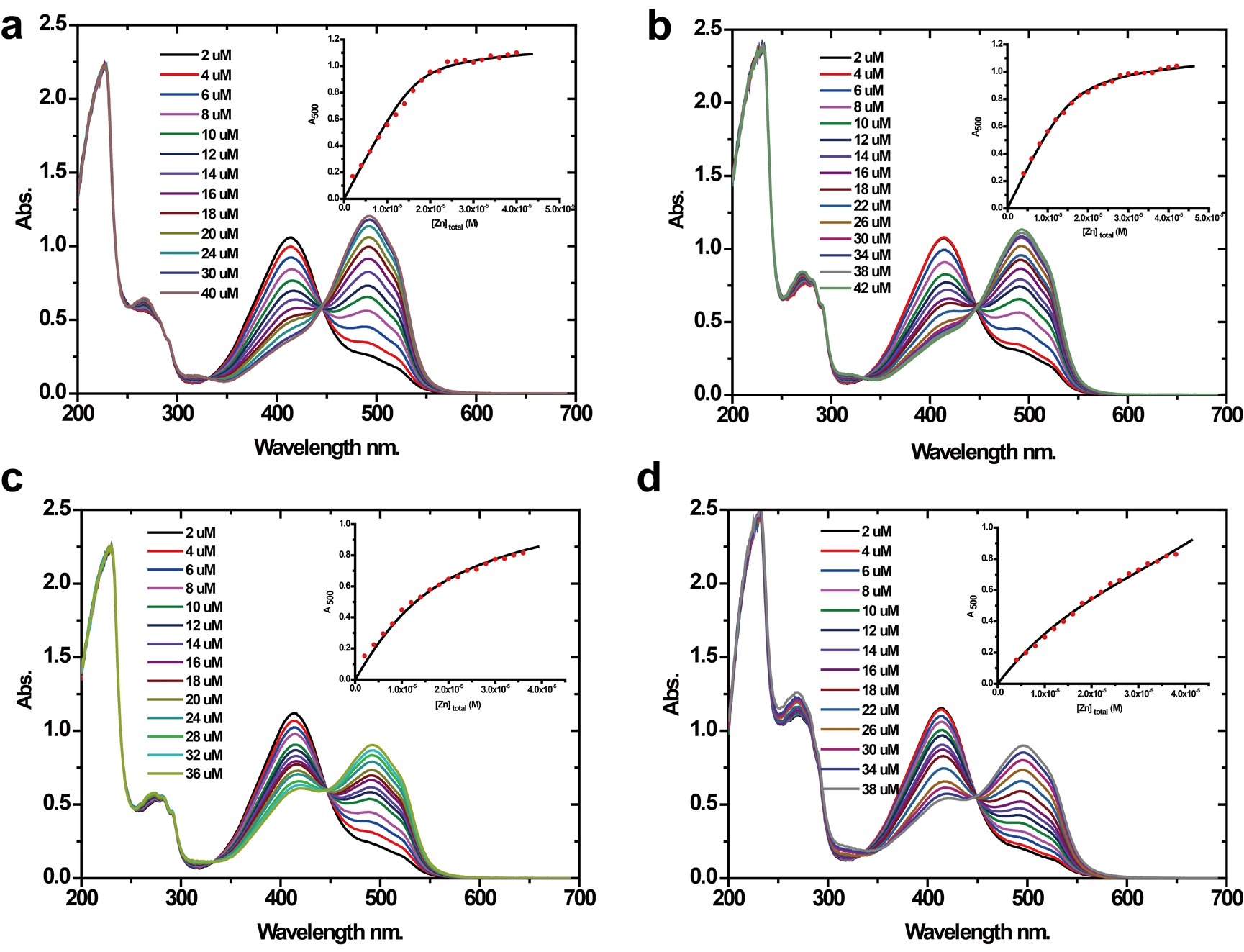

Supplement: S6 Fig — The representative titration UV spectrum of PAR (36uM) with the increasing Zn(II) (2 uM-42 uM) was recorded in the presence of (a) 68 uM CzcS SD, (b) 105 uM CzcS SD H55C, (c) 60 uM CzcS SD D60C, and (d) 72 uM CzcS SD L38C H55A competition in the range of 200 nm to 700 nm. The titration data at 500 nm and fitting binding isotherm by using Dynafit software [30] were inserted in corresponding titration UV spectrum. (TIF) [file ppat.1006533.s006.tif]

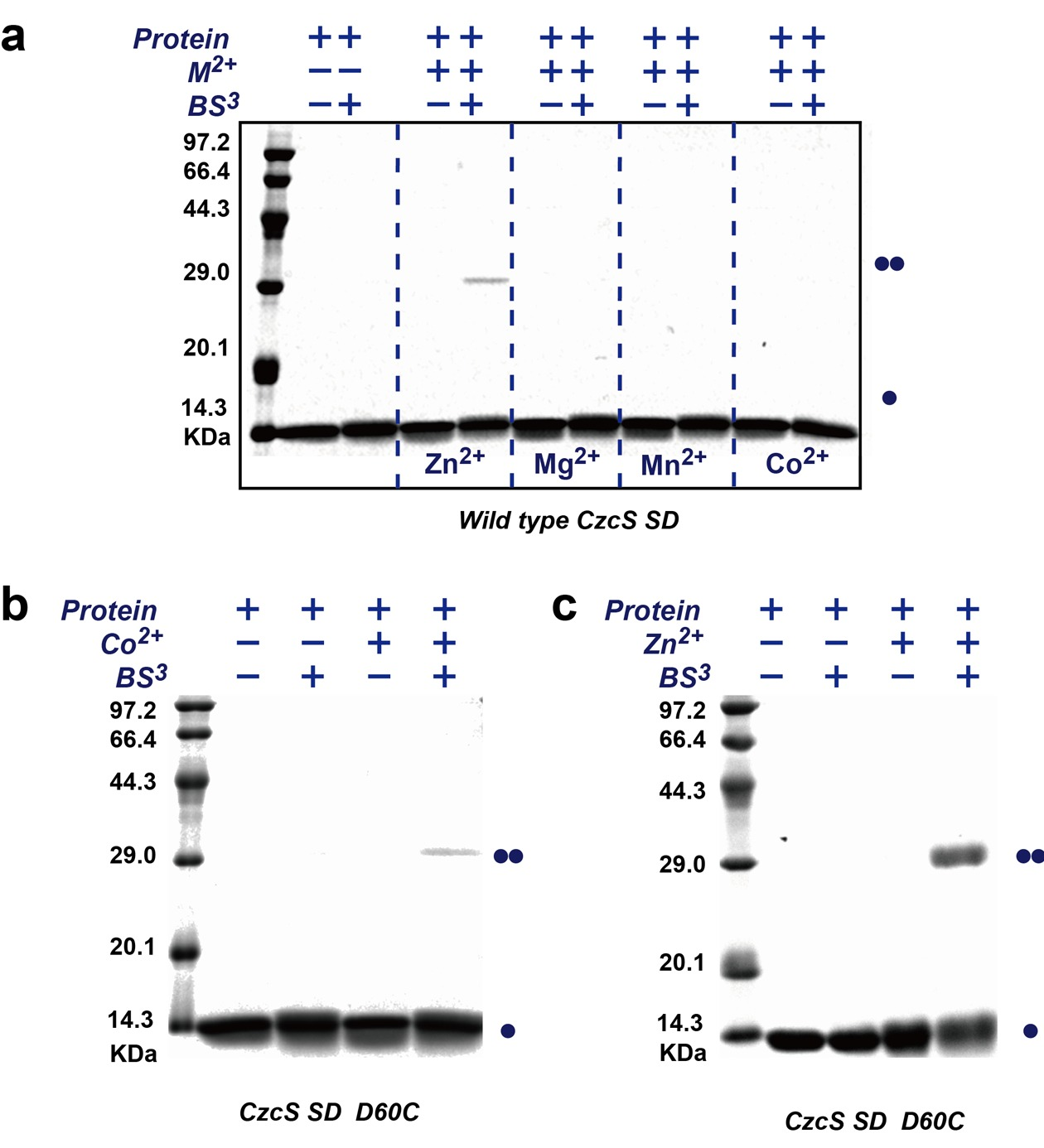

Supplement: S7 Fig — The monomer and dimer of wild type and mutant CzcS SD are indicated with ● and ●●, respectively. (a) The Zn(II) induced crosslinked dimerization of wild type CzcS SD. Other divalent cations such as Mg(II), Co(II), and Mn(II) in the experiments are supplied as negative control. (b) The Co(II) induced crosslinked dimerization of CzcS SD D60C mutant. (c) The Zn(II) induced crosslinked dimerization of CzcS SD D60C mutant. (TIF) [file ppat.1006533.s007.tif]

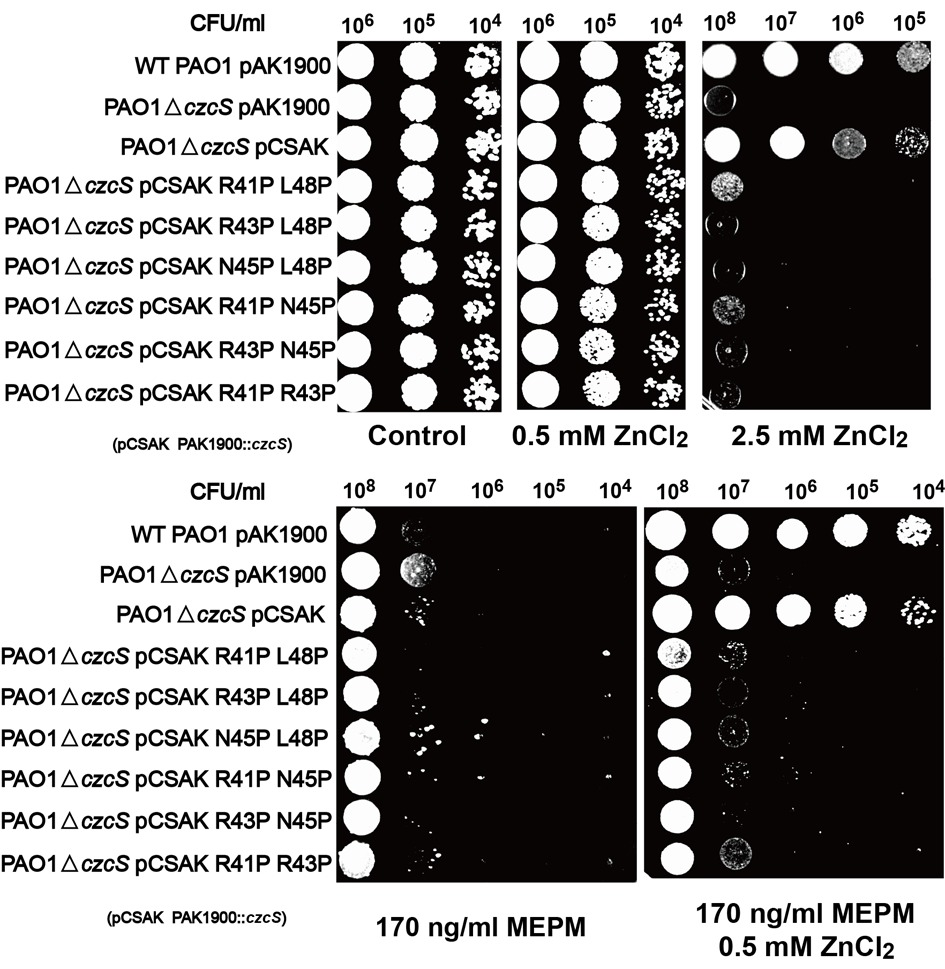

Supplement: S8 Fig — Wild type P. aeruginosa and its derivative strains are examined on the LB plates that contain Zn(II) and MEPM antibiotic as follows: wild type P. aeruginosa with the empty pAK1900 plasmid as the control (WT PAO1 pAK1900), czcS deficient P. aeruginosa with empty pAK1900 (PAO1△czcS pAK1900), czcS-deficient P. aeruginosa with wild type czcS encoded on pAK1900 (PAO1△czcS pCSAK), czcS-deficient P. aeruginosa complemented with czcS mutants in pAK1900 (PAO1△czcS pCSAK R41P L48P, PAO1△czcS pCSAK R43P L48P, PAO1△czcS pCSAK N45P L48P, PAO1△czcS pCSAK R41P N45P, PAO1△czcS pCSAK R43P N45P, and PAO1△czcS pCSAK R41P R43P). (TIF) [file ppat.1006533.s008.tif]

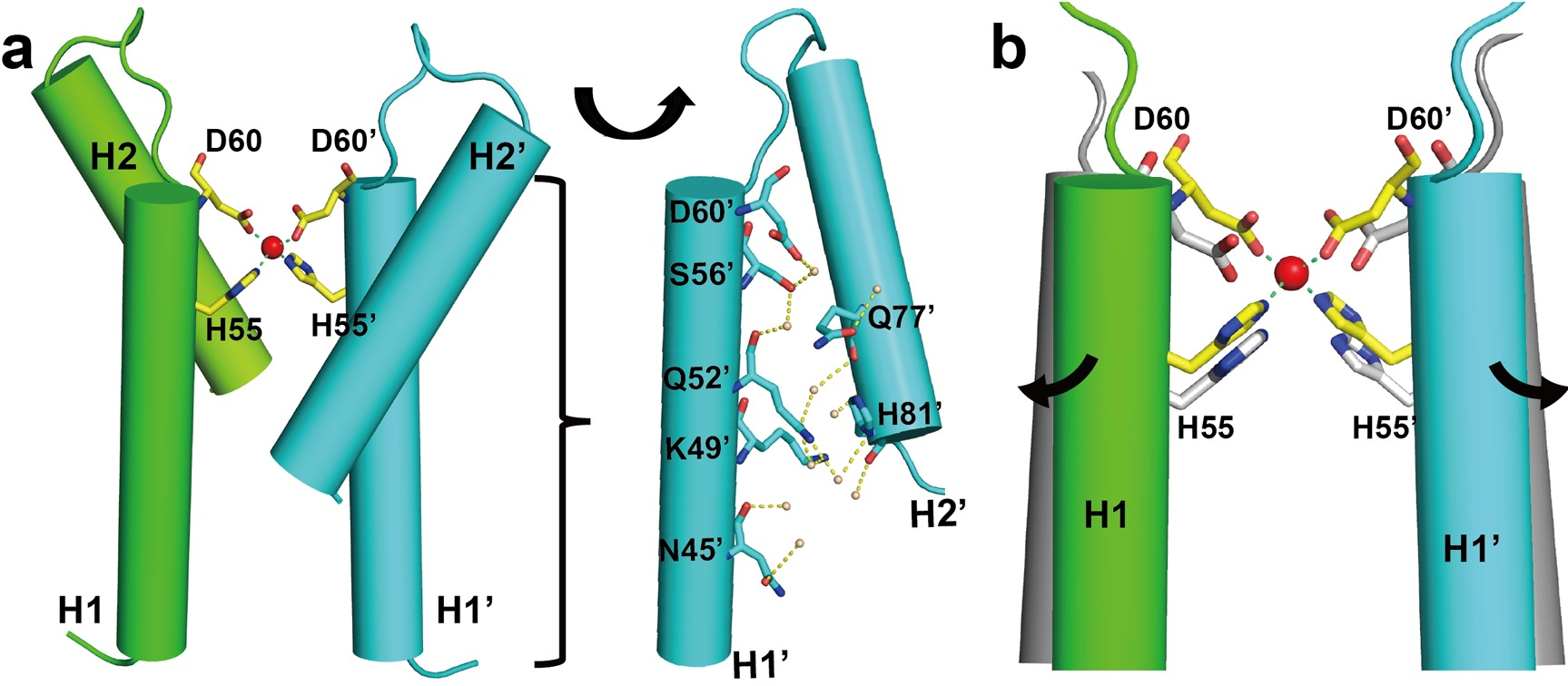

Supplement: S9 Fig — (a)The outer-shell environment of the H1 and H1’ α-helices. Other than the water molecule-mediated hydrogen-bond, there are no direct interactions between the residues of the H1 and H1’ α-helices. (b) Regulation of Zn(II) coordination geometry. A close-up view of the Zn(II) binding site confined between the H1 and H1’ α-helices (shown in cylinders). The black arrows indicate the hypothesized movements of the H1 and H1’ α-helices. (TIF) [file ppat.1006533.s009.tif]

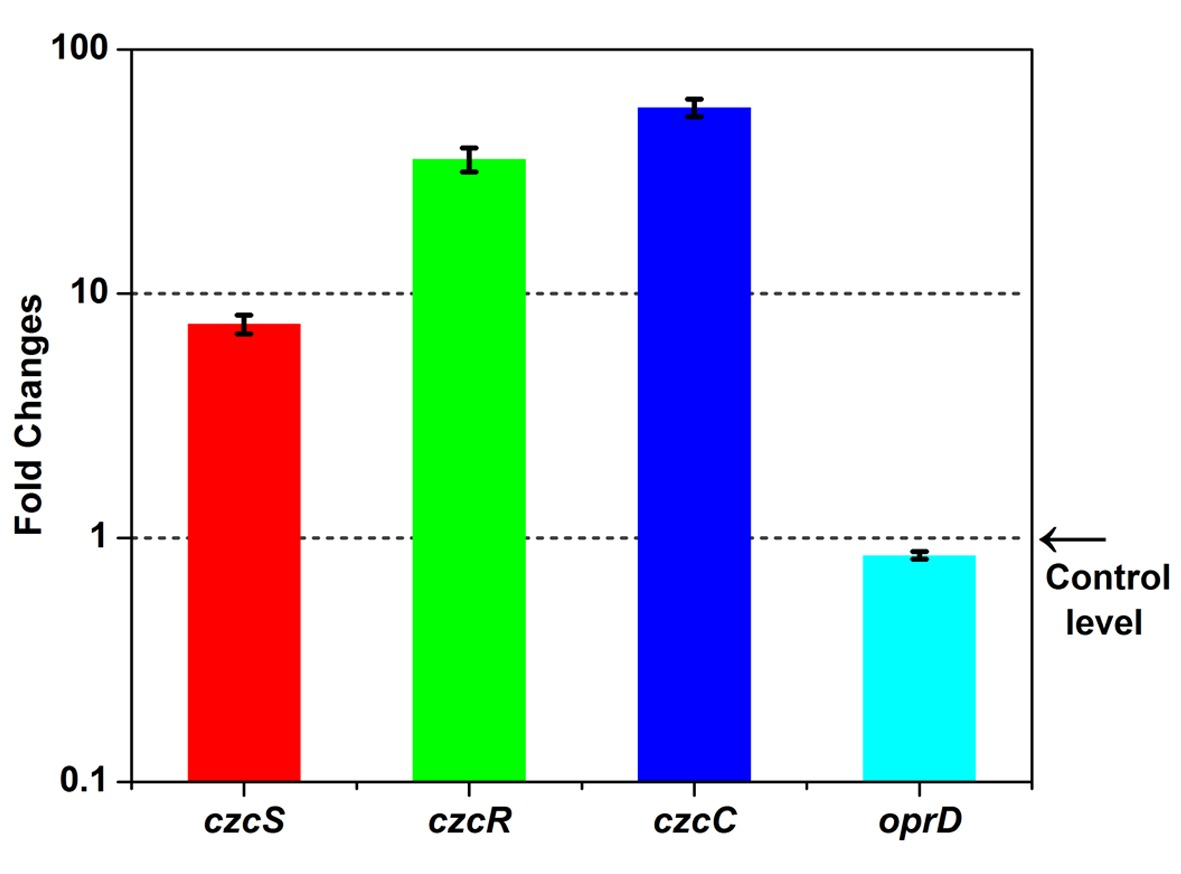

Supplement: S10 Fig — The experiments are performed in duplicate with the average results and standard deviations shown. (TIF) [file ppat.1006533.s010.tif]

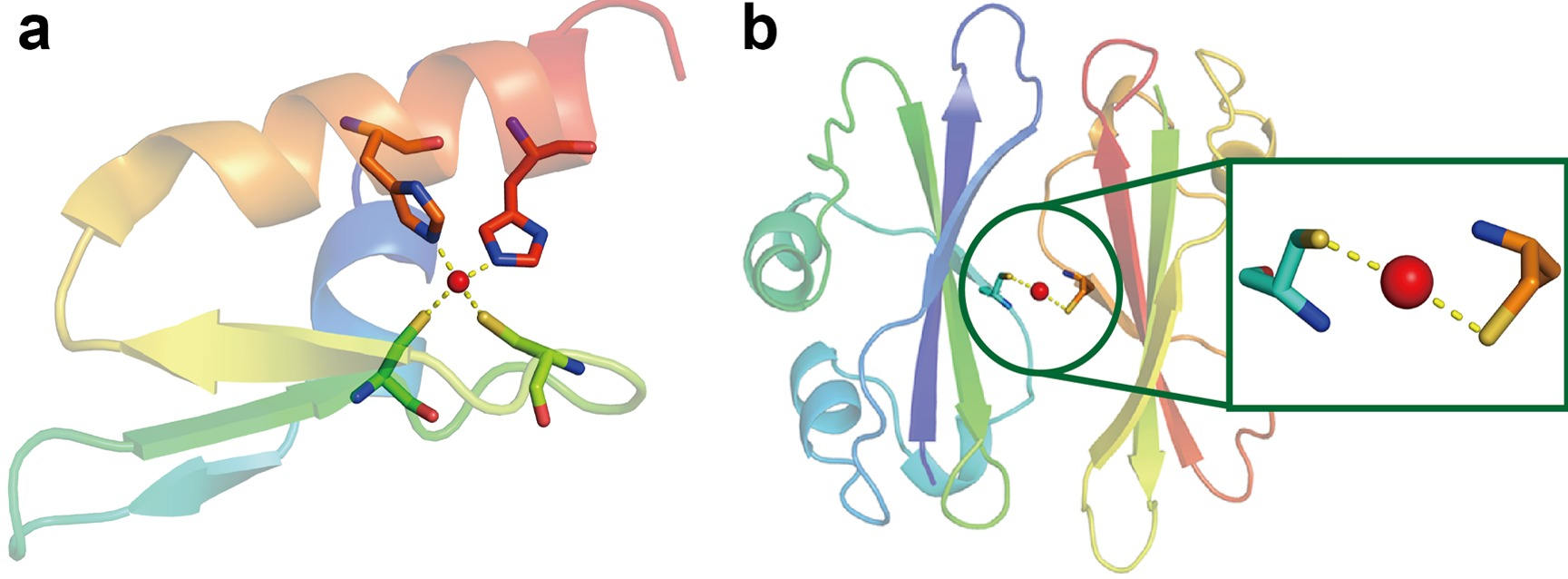

Supplement: S11 Fig — (a) In the D60C mutant, a tetrahedral coordination geometry analogy to the classic Cys2His2 zinc finger (PDB code: 1NCS) can be formed [43]. (b) In the H55C mutant, a linear coordination geometry can be formed between dithiolate and Zn(II), which is similar to that is formed on the dimer interface of the colicin E3 immunity protein from (PDB code: 3EIP) [44]. (TIF) [file ppat.1006533.s011.tif]

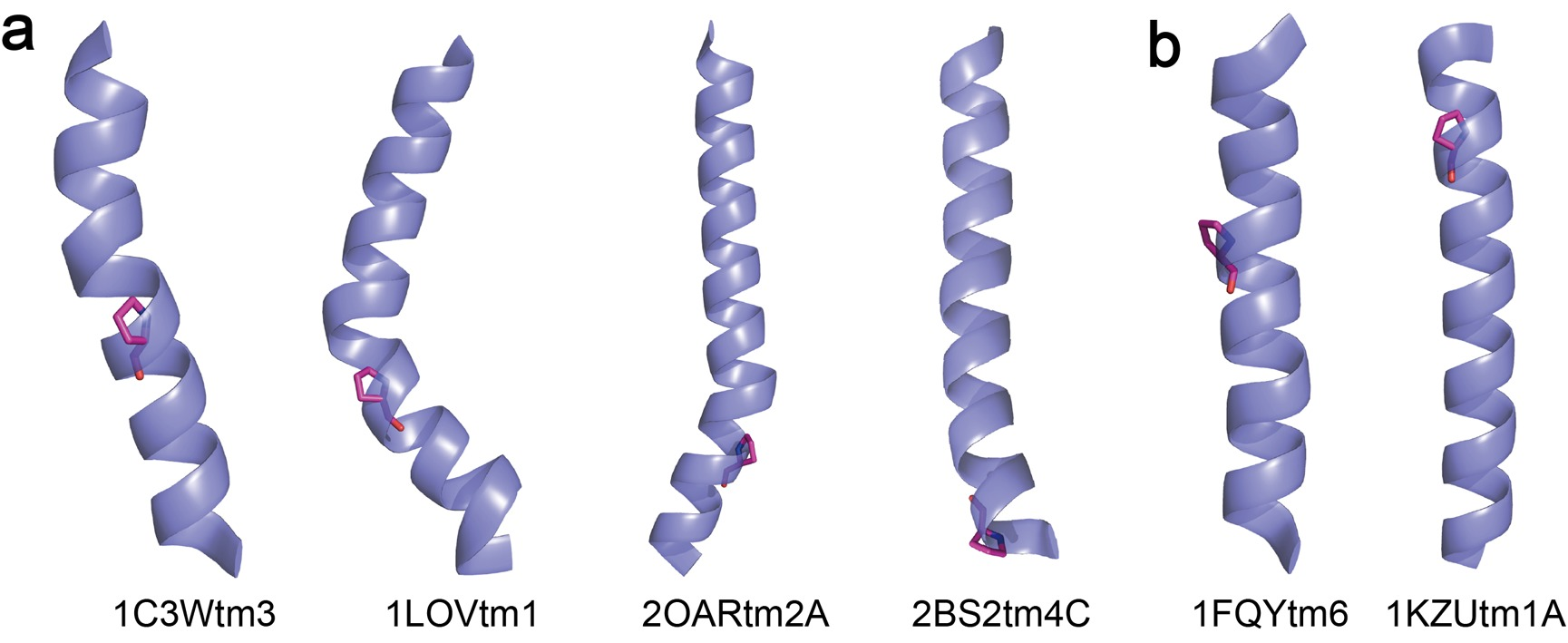

Supplement: S12 Fig — The helix is shown as cartoon in slate on which proline residue is shown as stick in magenta. (a) The proline-containing α-helices with obvious kink angle. (b) The proline-containing α-helices with no distortion. (TIF) [file ppat.1006533.s012.tif]
