# Supplementary material for: Structural basis of Zn(II) induced metal detoxification and antibiotic resistance by histidine kinase CzcS in Pseudomonas aeruginosa
Source: PLoS Pathog. 2017 Jul 21;13(7):e1006533. doi: 10.1371/journal.ppat.1006533 (PMC5540610; doi:10.1371/journal.ppat.1006533)
Supplement: S1 Table — (DOC) [file ppat.1006533.s013.doc]

**S1 Table. The model parameters analyzed by DynaFit in determining the dissociation constants of wild type and mutant CzcS SD with Zn(II)**

|  | **Fitted^[a]^** | **Std. Error^[b]^** | **CV(%)^[c]^** |
| --- | --- | --- | --- |
| **K_d_*^CzcS SD^*** | 1.66396×10^-6^ | 2.4931×10^-7^ | 14.98 |
| **K_d_*^CzcS SD H55C^*** | 8.49283×10^-7^ | 3.76509×10^-8^ | 4.43 |
| **K_d_*^CzcS SD D60C^*** | 5.66891×10^-8^ | 2.53749×10^-9^ | 4.48 |
| **K_d_*^CzcS SD L38C H55A^*** | 9.43316×10^-9^ | 8.13638×10^-10^ | 8.63 |

**[a] The best fit values of dissociation constants analyzed by DynaFit from experimental data.**

**[b] The formal standard errors analyzed by DynaFit from experimental data.**

**[c] The coefficient of variation analyzed by DynaFit from experimental data.**
