# Supplementary material for: Structural basis of Zn(II) induced metal detoxification and antibiotic resistance by histidine kinase CzcS in Pseudomonas aeruginosa
Source: PLoS Pathog. 2017 Jul 21;13(7):e1006533. doi: 10.1371/journal.ppat.1006533 (PMC5540610; doi:10.1371/journal.ppat.1006533)
Supplement: S2 Table — (DOC) [file ppat.1006533.s014.doc]

**S2 Table. Non-conservative mutations of residues along the H1 and H1’ α-helices**

| **Mutants** | **Phenotype** | |
| --- | --- | --- |
|  | 2.5 mM Zn^2+^ | 170 ng/ml MEPM, 0.5 mM Zn^2+^ |
| Wild type | **+** | **+** |
| R41K | **+** | **+** |
| R41F | **+** | **+** |
| R43E | **+** | **+** |
| N45D  N45F  L48I  L48F | **+**  **+**  **+**  **+** | **+**  **+**  **+**  **+** |

**+: The mutation strains display equivalent wild type activities in sensing and regulating the zinc signal.**
