# Supplementary material for: Structural basis of Zn(II) induced metal detoxification and antibiotic resistance by histidine kinase CzcS in Pseudomonas aeruginosa
Source: PLoS Pathog. 2017 Jul 21;13(7):e1006533. doi: 10.1371/journal.ppat.1006533 (PMC5540610; doi:10.1371/journal.ppat.1006533)
Supplement: S3 Table — (DOC) [file ppat.1006533.s015.doc]

**S3 Table. Oligonucleotides used in this study**

| **Name** | **Sequence (5’ 3’)** |
| --- | --- |
| primers I | AT**GAATTC**CTGGCGCTTCTCCAGGTC |
| primers II | CG**TCTAGA**AGCGACAGTCGGCCTGGT |
| primers III | GC**TCTAGA**GGTACTCGCCGCAGAAAC |
| primers IV | GC**AAGCTT**CCACTGGGTCAACCAGAAAC |
| pCSAK I | GTAGT**AAGCTT**GTTCCGCTCCTCGT |
| pCSAK II | GGCCTGGTGGCGTCATGTTCGCCCCTATATAA |
| pCSAK III | TTATATAGGGGCGAACATGACGCCACCAGGCC |
| pCSAK IV | GTAGT**GGATCC**TCAGGCAGCGCCCGTC |
| L38Cf | CGCTCGCGGGCTTCACACTCGCGGCCGAGGT |
| L38Cr | ACCTCGGCCGCGAGTGTGAAGCCCGCGAGCG |
| R41Pf | GTTTTCCCGCTCGGGGGCTTCCAGCTC |
| R41Pr | GAGCTGGAAGCCCCCGAGCGGGAAAAC |
| R41Kf | GCAGGTTTTCCCGCTCTTTGGCTTCCAGCTCGCGG |
| R41Kr | CCGCGAGCTGGAAGCCAAAGAGCGGGAAAACCTGC |
| R41Ff | GCAGGTTTTCCCGCTCAAAGGCTTCCAGCTCGCGG |
| R41Fr | CCGCGAGCTGGAAGCCTTTGAGCGGGAAAACCTGC |
| R43Pf | ATTGCAGGTTTTCCGGCTCGCGGGCTTC |
| R43Pr | GAAGCCCGCGAGCCGGAAAACCTGCAAT |
| R43Ef | TCAATTGCAGGTTTTCCTCCTCGCGGGCTTCCAGC |
| R43Er | GCTGGAAGCCCGCGAGGAGGAAAACCTGCAATTGA |
| N45Pf | AGCCCGCGAGCGGGAACCCCTGCAATTGAAGC |
| N45Pr | GCTTCAATTGCAGGGGTTCCCGCTCGCGGGCT |
| N45Df | TCAATTGCAGGTCTTCCCGCTCGCGGGC |
| N45Dr | GCCCGCGAGCGGGAAGACCTGCAATTGA |
| N45Ff | GCTTCAATTGCAGGAATTCCCGCTCGCGGGCT |
| N45Fr | AGCCCGCGAGCGGGAATTCCTGCAATTGAAGC |
| L48Pr | AGCGGGAAAACCTGCAACCGAAGCTGGAGCAGATCC |
| L48If | GGATCTGCTCCAGCTTTATTTGCAGGTTTTCCCGCTC |
| L48Ir | GAGCGGGAAAACCTGCAAATAAAGCTGGAGCAGATCC |
| L48Ff | TGCTCCAGCTTGAATTGCAGGTTTTCCCGCTC |
| L48Fr | GAGCGGGAAAACCTGCAATTCAAGCTGGAGCA |
| L50Pf | GGCGGATCTGCTCCGGCTTCAATTGCAGG |
| L50Pr | CCTGCAATTGAAGCCGGAGCAGATCCGCC |
| Q52Pf | GGCTGTGGCGGATCGGCTCCAGCTTCAAT |
| Q52Pr | ATTGAAGCTGGAGCCGATCCGCCACAGCC |
| H55Af | CGTCTTCCAGGCTGGCGCGGATCTGCTCCA |
| H55Ar | TGGAGCAGATCCGCGCCAGCCTGGAAGACG |
| H55Cf | TCGTCTTCCAGGCTGCAGCGGATCTGCTCCAG |
| H55Cr | CTGGAGCAGATCCGCTGCAGCCTGGAAGACGA |
| H55Rf | GAGCAGATCCGCCGCAGCCTGGAAGAC |
| H55Rr | GTCTTCCAGGCTGCGGCGGATCTGCTC |
| D60Af | GCGCAGGTCGAGAGCGTCTTCCAGGCT |
| D60Ar | AGCCTGGAAGACGCTCTCGACCTGCGC |
| D60Cf | CTGCGCAGGTCGAGACAGTCTTCCAGGCTGTG |
| D60Cr | CACAGCCTGGAAGACTGTCTCGACCTGCGCAG |
| H72Af | GTCCTGCAGGGCAGCCGCCTGTACGGCC |
| H72Ar | GGCCGTACAGGCGGCTGCCCTGCAGGAC |
| D76Af | CGCCACCAGTTGGGCCTGCAGGGCATG |
| D76Ar | CATGCCCTGCAGGCCCAACTGGTGGCG |
| *rpsL*RTF | GCAACTATCAACCAGCTGGTG |
| *rpsL*RTR | GCTGTGCTCTTGCAGGTTGTG |
| *czcS* RTF | TACGCCAGCTCTCGCAGTTCTCC |
| *czcS* RTR | TGTCCACCTGCACCAGGAACAGC |
| *czcR* RTF  *czcR* RTR | GTCATCACCCGGACGCAGATCAT  GTAGCCGACGCCGCGAATGGTAT |
| *czcC* RTR | CGAGGTGGCCGATGTCTT |
| *oprD* RTF | CTGGGCTTCGATTTCAACAT |
| *oprD* RTR | GCCGAAGCCGATATAATCAA |
| **The restriction sites were shown as bold** | |
